# Is the developmental stage important for the plasticity of transgenerational immune priming?

**DOI:** 10.1242/bio.062421

**Published:** 2026-08-27

**Authors:** Fernanda Vázquez-Fuerte, Angélica González-Hernández, Juan Carlos Torres-Guzmán, Jorge Contreras-Garduño

**Affiliations:** ^1^ Edificio de Investigación I, ENES, Unidad Morelia, UNAM, Antigua Carretera a Pátzcuaro No. 8701. Col. Ex-Hacienda San José de la Huerta, Código Postal 58190, Morelia, Michoacán, Mexico; ^2^ Departamento de Biología, División de Ciencias Naturales y Exactas, Universidad de Guanajuato, Noria Alta s/n, Guanajuato 36050, Estado de Guanajuato, Mexico

**Keywords:** Immune memory, Immune training, Trained immunity, Ecoimmunology, *Spodoptera*, Trade-offs

## Abstract

Parental transfer of immune memory can increase offspring survival, although its benefits may vary across developmental stages. We investigated transgenerational immune priming in the fall armyworm (*Spodoptera frugiperda*) infected with the fungus *Metarhizium brunneum*. Using a split design, fathers and mothers were exposed to a low-dose fungal challenge at larval stages L5 or L6, and their offspring were subsequently challenged with a high dose at L5 or L6. We assessed survival, development, immune responses (phenoloxidase, lytic activity), and oxidative stress (total antioxidant capacity, catalase, superoxide dismutase, hydrogen peroxide). Parental immune activation at either instar had no significant effect on offspring survival. However, offspring responses were stage dependent: L5 larvae in the memory group (homologous challenge) showed higher survival than heterologous or L6-challenged larvae. No L6-challenged larvae reached adulthood. The L5 memory group developed more slowly than controls, while all L6 larvae failed to complete development. Immune response and oxidative stress were higher in L6 larvae, but immune priming effects did not differ. These results suggest that developmental constraints and self-inflicted damage from immune and oxidative responses limit immune priming efficiency at later stages. Our findings highlight that transgenerational immune priming is strongly influenced by the developmental stage at which offspring are challenged.

## Teaser

**Summary:** Parental transfer of immune memory to offspring is crucial for their survival. However, not all offspring have equal opportunities to benefit from this protection, particularly if the developmental stage of offspring is a key determinant of survival.

## INTRODUCTION

Insects have successfully proliferated across diverse habitats, a feat often attributed to their robust immune defenses that mitigate the constant pressure of pathogenic infection (Melillo et al., 2018). Although invertebrate immunity was long considered purely innate (Melcarne et al., 2019), accumulating evidence demonstrates that many taxa exhibit different forms of immune memory (Pradeu and Du Pasquier, 2018; Kurtz et al., 2025). Specifically, immune priming enables invertebrates exposed to sublethal or inactivated pathogens to mount stronger responses, resulting in higher survival and reduced infection upon subsequent challenge; when this protection is directed at specific pathogen species or strains, it is termed specific immune memory (Kurtz and Franz, 2003; Little and Kraaijeveld, 2004; Contreras-Garduño et al., 2016; Milutinovic and Kurtz, 2016; Lanz-Mendoza and Garduño, 2018; Kurtz et al., 2025).

The immune priming protection can extend across generations when parents and offspring share the same environment and are exposed to similar parasites (Pigeault et al., 2016). This phenomenon, termed transgenerational immune priming (TGIP), occurs when parental exposure to pathogens confers protective benefits to their offspring (Moret, 2006). Despite these adaptive advantages, this form of immunity is evolutionarily costly and often incurs trade-offs in terms of growth, development, and reproductive potential (Metcalfe and Monaghan, 2001; Dubuffet et al., 2015; Tetreau et al., 2019; Tate and Graham, 2015; Lanz-Mendoza and Contreras-Garduño, 2022; Rutkowski et al., 2023; Ju et al., 2025). Examples include altered offspring immunity and life-history traits in mollusks (Yue et al., 2013) and reduced offspring numbers or delayed development in insects (Schulz et al., 2019; Roth et al., 2010). Developmental delays can reduce larval size and later reproductive success (Trauer and Hilker, 2013; Koella and Boëte, 2002). Despite these findings, it remains unclear how the parental developmental stage affects the immune information transmitted to offspring across their developmental stages and what costs this transmission entails (Tetreau et al., 2019).

There is indirect evidence supporting the previous hypothesis: within-generation immune priming has been linked to developmental timing. For instance, in *Tribolium castaneum*, protection against *Bacillus thuringiensis* depended on whether priming occurred at larval versus adult stages (Khan et al., 2016). Different life stages experience distinct ecological niches, pathogen exposures, and trade-offs in immune investment, and theoretical models predict stage-dependent susceptibility (Boots, 2004; Tate and Rudolf, 2012). Consequently, immune priming may be hard to detect when parents are challenged near energetically demanding transitions (e.g. late larval instars approaching pupation) compared to those whose development is far from the demanding transitions (larval instars not approaching pupation). To address these gaps, we propose to investigate how parental developmental stages that are far or near pupation affect TGIP in *Spodoptera frugiperda*, a globally distributed polyphagous pest frequently exposed to entomopathogenic fungi such as *Metarhizium* in agroecosystems (Deshmukh et al., 2018; Goergen et al., 2016; Molina-Ochoa et al., 2003; Pogue, 2002; Wan et al., 2021). *Metarhizium* infects via the cuticle and is widely used as a biological control agent (Perumal et al., 2023; Ortiz-Urquiza and Keyhani, 2013), making this system well suited to examine the ecological and evolutionary consequences of stage-dependent immune priming.

*S. frugiperda* undergoes six larval instars before pupation (Schmidt-Duran et al., 2015), providing a convenient framework to test stage-dependent effects of immune priming. We focused on the fifth and sixth instars because these late stages immediately precede metamorphosis, when trade-offs between continued growth, developmental transitions and immune investment are likely intensified. Indirect evidence is consistent with this interpretation, as fifth-instar larvae have been reported to exhibit a stronger immune response than sixth-instar larvae (Wang et al., 2025). Individuals at the sixth instar may reallocate resources away from immune defense in preparation for pupation. Studies in related species (e.g. *Spodoptera littoralis* and *Spodoptera exempta*) report that transgenerational protection depends on rearing context and pathogen dose, underscoring the context dependence of costs and benefits (Wilson and Graham, 2015; Wilson et al., 2021). We therefore tested whether parental priming at the fifth versus the sixth instars differentially affects offspring resistance to fungal challenge.

Invertebrate defense is a coordinated network of cellular and humoral effectors that contribute to pathogen control while risking self-damage if left unchecked (Dupuy et al., 1991; Fridovich, 2004; Browne et al., 2013; Sugumaran and Barek, 2016; Ragland and Criss, 2017; Huan et al., 2020; Mookherjee et al., 2020). For example, entomopathogenic fungi provoke a suite of host responses, including activation of the phenoloxidase cascade, melanization, and redox-related pathways (Marmaras et al., 1996; Duffield et al., 2023). Fungal infection also modulates antioxidant enzymes such as catalase (CAT) and superoxide dismutase (SOD), highlighting the central role of redox balance in host–pathogen interactions (Zug and Hammerstein, 2015; Perumal et al., 2023; Kodrík et al., 2015). To capture this integrative immune state in our study, we measured phenoloxidase (PO) activity, lytic (antimicrobial) activity, total antioxidant capacity (TAC), CAT and SOD activities, and hydrogen peroxide (H_2_O_2_) levels as complementary indicators of immune competence and oxidative balance during TGIP.

## RESULTS

### Activation of the parental immune response

No significant differences were found in survival to adulthood between the parental groups primed in L5 and L6 (χ^2^=1.21, d.f.=1, *P*=0.27). Regarding development, no significant differences were found between treatments in the L5 and L6 stages in the probability of pupating (χ^2^=0.87, d.f.=1, *P*=0.35) or in the probability of reaching the adult phase (χ^2^=0.32, d.f.=1, *P*=0.57). This suggests that immune activation (priming) was not costly to parents.

### Effect of TGIP on survival

**Fig. 1.**
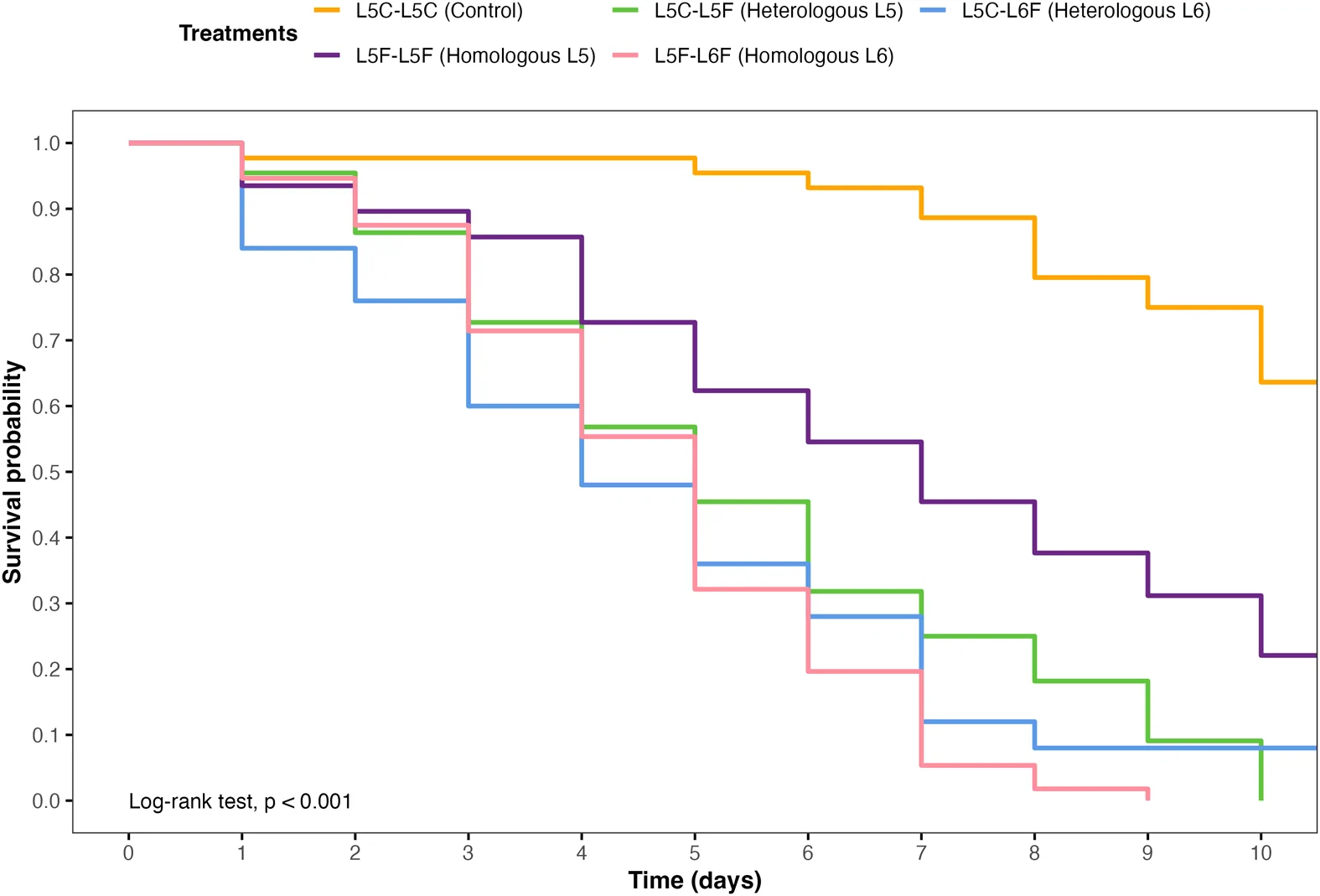
**Kaplan–Meier survival curves of offspring from parents primed at the L5 stage according to experimental treatments.** In the labels, F denotes individuals challenged with the fungus, and C denotes untreated controls. Offspring were assigned to the following treatments: control (L5C–L5C, *n*=44), in which both parents and offspring were injected with Tween 80; homologous L5 (L5F–L5F, *n*=77), in which both parents and offspring were challenged at the L5 stage; heterologous L5 (L5C–L5F, *n*=44), in which parents were controls and offspring were challenged at the L5 stage; homologous L6 (L5F–L6F, *n*=56), in which parents were challenged at the L5 stage and offspring at the L6 stage; and heterologous L6 (L5C–L6F, *n*=25), in which parents were controls and offspring were challenged at the L6 stage. The *y*-axis represents the estimated survival probability calculated using the Kaplan–Meier estimator. Differences among survival curves were evaluated using log-rank tests.

We found significant differences in survival between treatments in the offspring from the parents primed in the L5 stage (χ^2^=111, d.f.=4, *P*<0.001). The control group (L5C–L5C) exhibited the highest survival probability (S_10_≈0.64), which was significantly greater than that observed in all challenged treatments (*P*<0.001 in all comparisons). Survival in the control group differed only marginally from that of the homologous L5 group (L5F–L5F: S_10_≈0.22; *P*=0.05). Among challenged offspring groups, the homologous L5 treatment showed a significantly higher survival probability than the heterologous L5 treatment (L5C–L5F: S_10_=0; *P*=0.006) as well as both homologous and heterologous treatments challenged at the L6 stage (L5F–L6F: S_10_=0 and L5C–L6F: S_10_=0.08; *P*<0.001). In contrast, no significant differences were found between the heterologous L5 treatment and the homologous or heterologous L6 treatment (*P*=0.99); these L6 treatments exhibited the lowest survival probabilities by day 10 (Fig. 1). These results indicate that TGIP in terms of survival occurs only when both parents and offspring are challenged in the L5 stage.

**Fig. 2.**
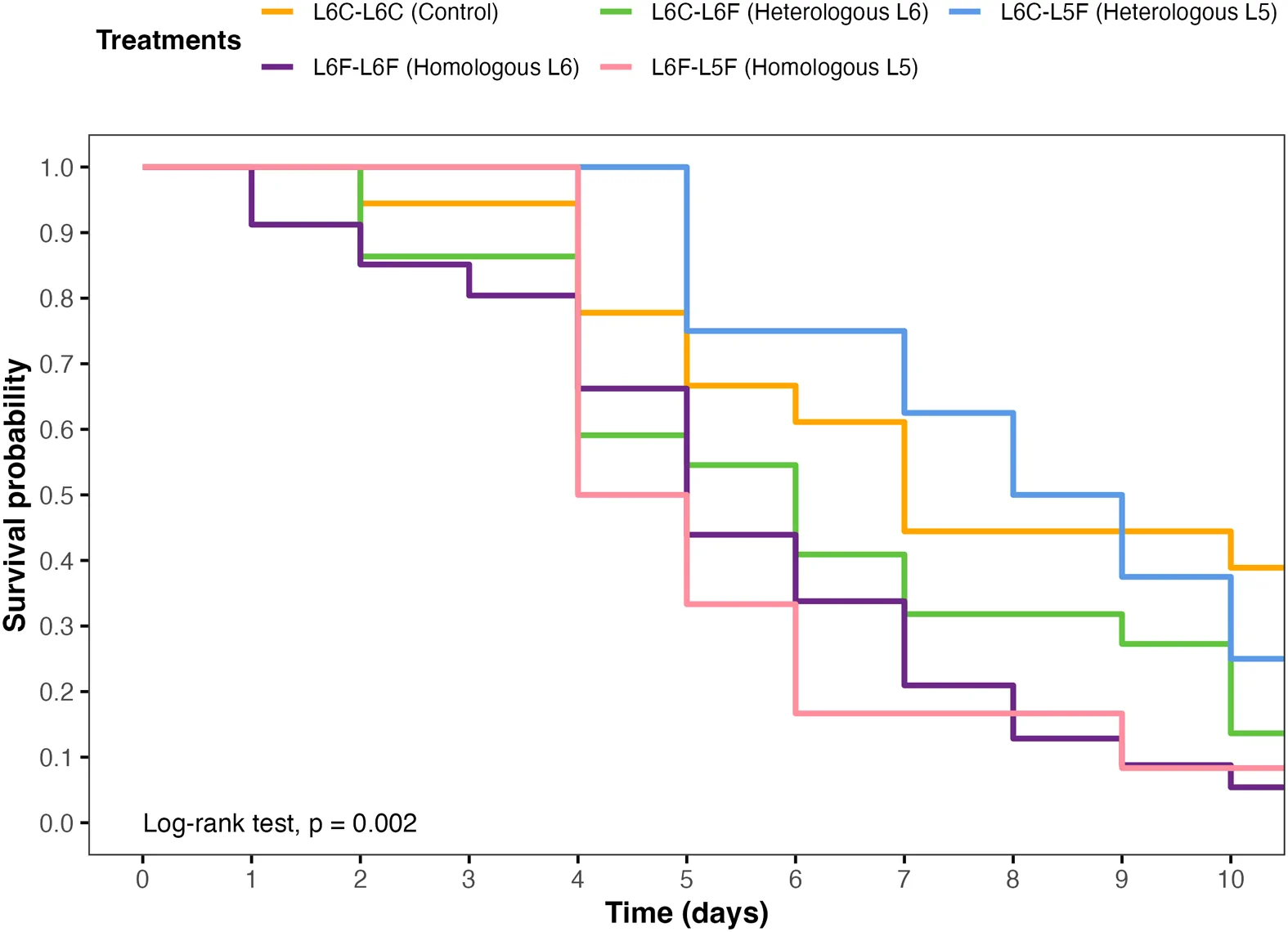
**Kaplan–Meier survival curves of offspring from parents primed at the L6 stage according to experimental treatments.** In the labels, F denotes individuals challenged with the fungus, and C denotes untreated controls. Offspring were assigned to the following treatments: control (L6C–L6C, *n*=18); heterologous L5 (L6C–L5F, *n*=8); heterologous L6 (L6C–L6F, *n*=22); homologous L5 (L6F–L5F, *n*=12); and homologous L6 (L6F–L6F, *n*=148). Differences among survival curves were evaluated using log-rank tests.

Survival probability differed significantly among treatments in offspring from parents primed at the L6 stage (log-rank test: χ^2^=17.0, d.f.=4, *P*=0.002). This overall effect was driven by the control group (L6C–L6C: S_10_≈0.39), which showed a significantly higher survival probability than the homologous L6 treatment (L6F–L6F: S_10_≈0.05; *P*=0.008). In contrast, no significant differences were found between the control group and the heterologous treatments (L6C–L5F: S_10_≈0.25 and L6C–L6F: S_10_≈0.14; *P*>0.11), nor among the challenged treatments themselves (*P*>0.33; Fig. 2). Overall, these results indicate that parental challenge at the L6 stage does not confer a survival advantage to the offspring, suggesting that immune priming at this stage does not enhance resistance.

When comparing the homologous treatment at the L5 stage from parents primed in the L5 stage and the homologous treatment at the L6 stage from parents primed in the L6 stage, the homologous treatment at the L5 stage had a higher probability of survival than the offspring challenged in the L6 stage (χ^2^=9.51, d.f.=1, *P*=0.002; Figs 1 and 2). Together, this evidence suggests that the stage at which both the parents and the offspring are challenged impacts the outcome of the TGIP, favoring memory at the L5 stage.

### Effect of TGIP on development

**Fig. 3.**
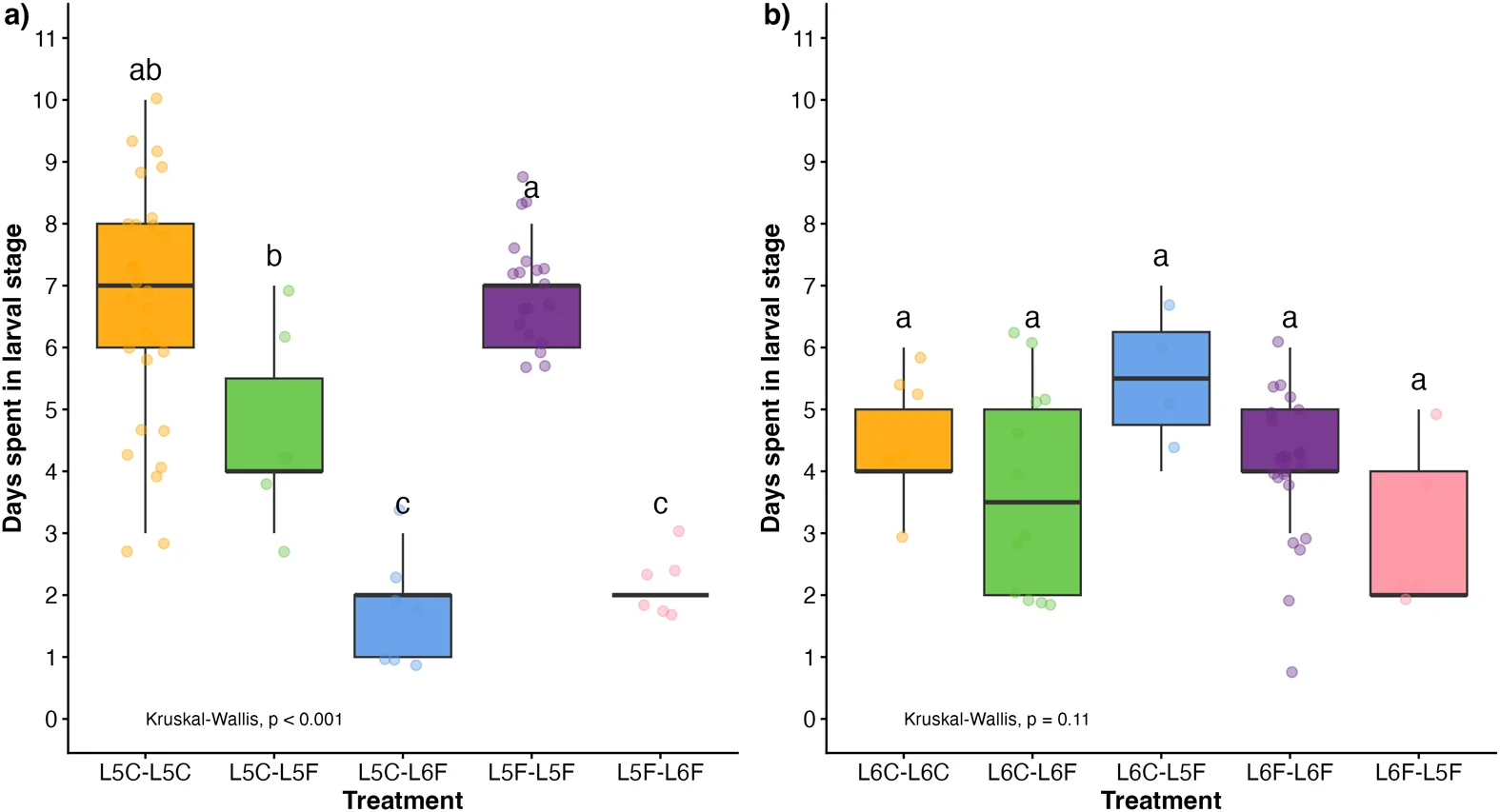
**Developmental time of offspring from parents primed at the L5 or L6 stages.** (A,B) Boxplots show the number of days individuals spent in the larval stage for offspring from parents challenged at the L5 stage (A) and offspring from parents challenged at the L6 stage (B). Boxes represent the median and interquartile range, whiskers indicate 1.5× the interquartile range, and points represent individual larvae. Different letters indicate significant differences among treatments within each panel, based on Kruskal–Wallis tests followed by pairwise Wilcoxon rank-sum tests with Bonferroni correction (*P*<0.05). Groups are color-coded as shown in the figure. Sample sizes were as follows: (A) L5C–L5C (*n*=30), L5C–L5F (*n*=6), L5C–L6F (*n*=7), L5F–L5F (*n*=19), and L5F–L6F (*n*=6); (B) L6C–L6C (*n*=7), L6C–L6F (*n*=12), L6C–L5F (*n*=4), L6F–L6F (*n*=24), and L6F–L5F (*n*=5).

Parental priming significantly affected larval offspring developmental time (χ^2^=36.17, d.f.=4, *P*=2.67×10^−7^, Fig. 3). Within treatments challenged at the L5 stage, larval developmental time differed between heterologous and homologous treatments, with larvae from the heterologous treatment (L5C–L5F=4.66±1.5 days, Fig. 3A) reaching pupation significantly faster than those from the homologous treatment (L5F–L5F=6.95±0.85 days; *P*=0.04). Neither treatment differed significantly from the control group (*P*>0.26; L5C–L5C=6.63±1.85 days). In contrast, larvae challenged at the L6 stage, regardless of whether the challenge was heterologous (L5C–L6F=1.71±0.76 days) or homologous (L5F–L6F=2.17±0.41 days), reached pupation significantly faster than all treatments challenged at the L5 stage (*P*<0.01). Consequently, no significant difference was identified between heterologous (L5C–L6F) and homologous (L5F–L6C) groups at the L6 stage (*P*=1.0; Fig. 3A).

At the L6 stage, no significant differences were found in the duration of larval development in offspring (Fig. 3B; χ^2^=7.48, d.f.=4, *P*=0.11; L6C–L6C=4.43±0.98 days; L6F–L6F=4.0±1.06 days; L6C–L6F=3.75±1.60 days; L6F–L5F=3.0±1.41 days; L6C–L5F=5.5±1.29 days). When homologous and heterologous treatments are compared for those infected at the L5 and L6 stages, larval developmental time differed significantly among treatments (χ^2^=36.3, d.f.=3, *P*<0.001, Fig. 3). The heterologous treatment challenged at the L5 stage (L5C–L5F) did not differ from either homologous (L6F–L6F) or heterologous (L6C–L6F) treatments challenged at the L6 stage (*P*=1.0). These three treatments reached pupation significantly faster than the homologous L5 treatment (L5F–L5F, *P*<0.001). The results indicate that larval developmental time is influenced by both the developmental stage at which the offspring were challenged and the type of treatment.

At the pupal stage, no significant differences in duration were observed among treatments, either in offspring challenged at the L5 stage (χ^2^=0.02, d.f.=1, *P*=0.86) or in offspring challenged at the L6 stage (χ^2^=1.51, d.f.=1, *P*=0.21), or when directly comparing treatments across L5- and L6-challenged offspring (χ^2^=0.75, d.f.=1, *P*=0.38). Pupal duration was not influenced by parental priming or offspring challenge.

### Effect of TGIP on immune response

**Fig. 4.**
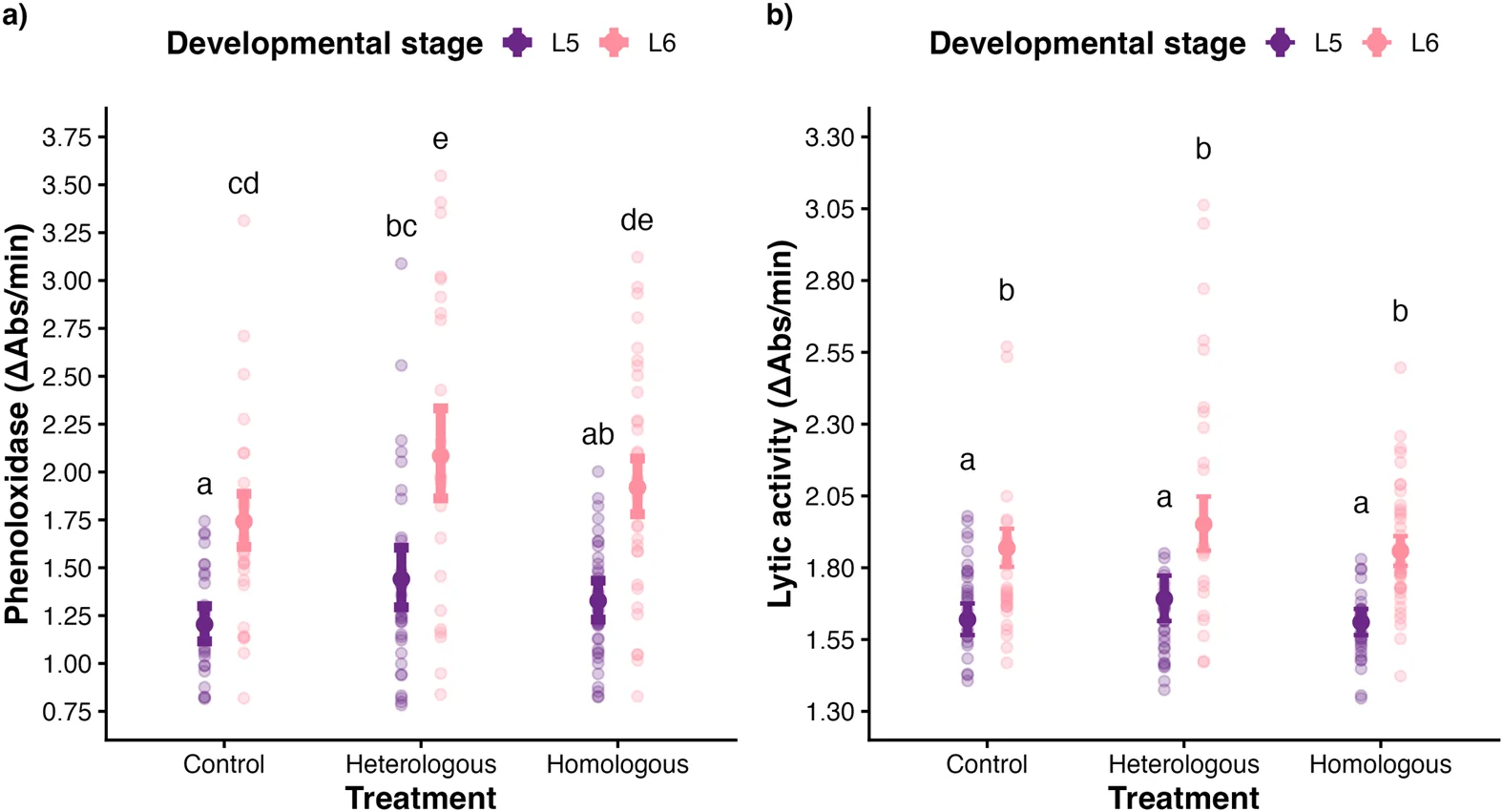
**Predicted immune response parameters in offspring at the L5 and L6 stages.** (A,B) Phenoloxidase (A) and lytic (B) activities. Points represent model-predicted means, and whiskers indicate 95% confidence intervals. Jittered points represent individual biological replicates. Colors denote the developmental stage at challenge (pink for L5 and purple for L6), while treatments are shown on the *x*-axis (control, homologous, and heterologous). Statistical analyses were performed using gamma GAMLSS, with developmental stage and treatment included as predictors of the mean response. Pairwise comparisons were conducted using estimated marginal means with Tukey-adjusted *P*-values. Different letters indicate significant differences among groups (*P*<0.05). Sample sizes were *n*=184 in total; L5: control L5C–L5C (*n*=31), heterologous L5C–L5F (*n*=31), homologous L5F–L5F (*n*=36); L6: control L6C–L6C (*n*=28), heterologous L6C–L6F (*n*=22), homologous L6F–L6F (*n*=36). The maximum rate of change in absorbance (*V*_max_) was used for subsequent analyses and expressed as ΔAbs/min.

For PO activity, the Akaike information criterion (AIC) showed that the most parsimonious model was the generalized additive model for location, scale, and shape (GAMLSS) with the dispersion parameter (*σ*) dependent on treatment (m_1_: AIC=243.38), compared to constant dispersion (m_0_: AIC=249.81) or dispersion dependent on the developmental stage (m_2_: AIC=247.07). The developmental stage had a significant effect on the mean PO activity, with higher values in L6 than in L5 across all treatments (Table S1). In addition, treatment significantly affected the dispersion parameter, with heterologous challenges associated with higher inter-individual variability in PO responses, while homologous challenges showed reduced dispersion compared to the control (Table S1). Pairwise comparisons revealed that PO activity was significantly higher in L6, both homologous (L6F–L6F) and heterologous (L6C–L6F), than in L5 homologous (L5F–L5F) and heterologous (L5C–L5F, all *P*<0.001). Across treatments, control individuals showed significantly lower values than heterologous-challenged individuals within both developmental stages (L5C–L5F and L6C–L6F; *P*=0.04), whereas homologous-challenged (L5F–L5F and L6F–L6F) individuals exhibited intermediate values that did not differ significantly from those of either group (*P*>0.29; Table S2; Fig. 4A). Extended analyses of estimated marginal means including the mixed developmental-stage treatments revealed that PO activity followed a developmental-stage gradient, with the highest values in L6 offspring (L6F–L6F and L6C–L6F), intermediate values in L5 (L5F–L5F and L6F–L6F), and the lowest values in the L5–L6 mixed group (L5C–L6F, L5F–L6F and L6C–L5F; Tables S3 and S4). The L6C–L5F mixed group exhibited intermediate PO activity and overlapped statistically with the L5C–L6F, L5F–L6F, and L5 groups (L5F–L5F and L5C–L5F), indicating that the developmental sequence between priming and challenge influenced the magnitude of the response. Although the fitted GAMLSS detected a small overall effect of treatment, homologous and heterologous groups within each developmental-stage combination were not significantly different by the Tukey-adjusted comparisons, suggesting that the developmental-stage combination explained more of the variation in PO activity than treatment identity in this extended analysis (Fig. S1A).

Regarding lytic activity, based on AIC, the most parsimonious model was the gamma GAMLSS with the dispersion parameter (*σ*) dependent on the developmental stage (m_2_: AIC=−69.45), compared to constant dispersion (m_0_: AIC=−28.54) or dispersion dependent on treatment (m_1_: AIC=−42.79). The developmental stage had a significant effect on both the mean and dispersion of lytic activity (Table S5). Pairwise comparisons revealed no significant differences in lytic activity among control, homologous, and heterologous treatments within L5 (L5C–L5C, L5F–L5F and L5C–L5F) or L6 (L6C–L6C, L6F–L6F and L6C–L6F) developmental stages (*P*>0.38). In contrast, lytic activity was consistently higher in L6 than in L5 across all treatments (all *P*<0.0001; Table S6; Fig. 4B). When mixed treatments were compared, the estimated marginal means also indicated that lytic activity was primarily determined by the developmental stage rather than treatment (Tables S7 and S8). The mixed developmental-stage groups, L5F–L6F, L5C–L6F and L6C–L5F, exhibited the lowest lytic activity and did not differ significantly from one another, regardless of whether the treatment was homologous or heterologous. In contrast, L5 offspring (L5F–L5F and L5C–L5F) showed significantly higher lytic activity than all mixed groups (L5F–L6F, L5C–L6F and L6C–L5F), whereas L6 offspring (L6F–L6F and L6C–L6F) exhibited the highest activity overall. Consistent with the GAMLSS results, homologous and heterologous treatments did not differ significantly in mean lytic activity (Table S7), and pairwise comparisons showed that homologous and heterologous groups within each developmental-stage combination shared the same Tukey grouping (Table S8). Together, these results indicate that lytic activity was driven primarily by the developmental stage, whereas treatment identity had no detectable effect on the response (Fig. S1B).

### Effect of TGIP on antioxidant defense

**Fig. 5.**
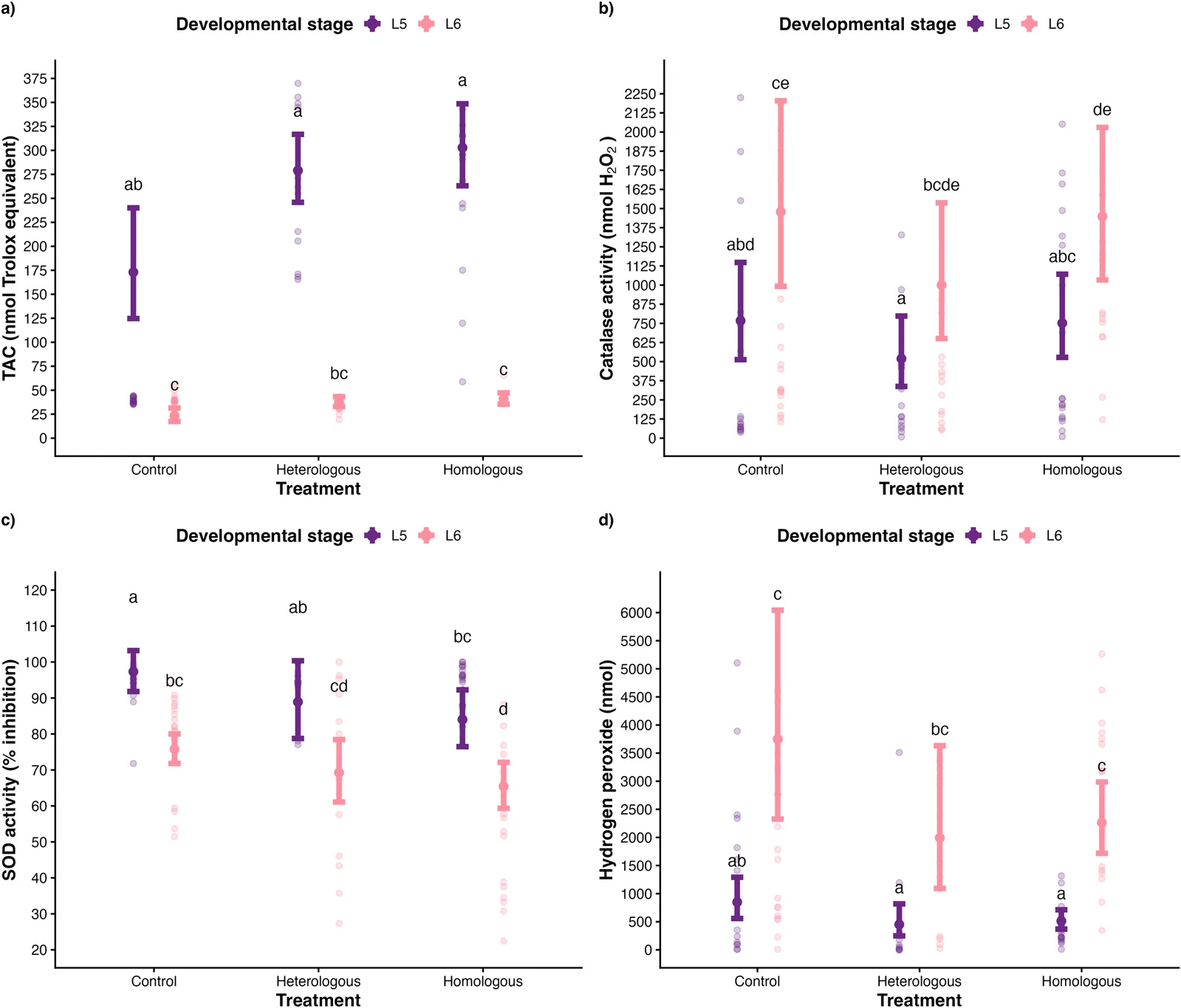
**Predicted antioxidant defense parameters in offspring at the L5 and L6 developmental stages.** (A–D) Total antioxidants (A), catalase (B), hydrogen peroxide (C), and SOD (D). Points represent model-predicted means, and whiskers indicate 95% confidence intervals. Jittered points represent individual biological replicates. Colors denote the developmental stage at challenge (pink for L5, purple for L6), while treatments are shown on the *x*-axis (control, homologous, and heterologous). Statistical analyses were performed using gamma GAMLSS, with developmental stage and treatment included as predictors of the mean response and, when appropriate, the dispersion. Pairwise comparisons were conducted using estimated marginal means with Tukey-adjusted *P*-values. Different letters indicate significant differences among groups (*P*<0.05). Sample sizes were *n*=184 in total; L5: control L5C–L5C (*n*=31), heterologous L5C–L5F (*n*=31), homologous L5F–L5F (*n*=36); L6: control L6C–L6C (*n*=28), heterologous L6C–L6F (*n*=22), homologous L6F–L6F (*n*=36).

For TAC, based on AIC, the most parsimonious model was the gamma GAMLSS with the dispersion parameter (*σ*) dependent on the developmental stage (m_2_: AIC=988.27), compared to constant dispersion (m_0_: AIC=1071.81) or dispersion dependent on treatment (m_1_: AIC=1040.95). The model showed that the developmental stage significantly affected both the mean and the dispersion of TAC. Specifically, the mean TAC was lower in L6 than in L5. Regarding the dispersion, the L6 stage also had significantly lower variability than L5 (Table S9). Pairwise comparisons indicated that TAC differed significantly between developmental stages, with all L5 treatments exhibiting higher TAC values than their corresponding L6 treatments (*P*<0.0001). Within both L5 and L6 stages, the control (L5C–L5C, L6C–L6C) group exhibited significantly lower TAC values than the challenged groups (homologous L5F–L5F and L6F–L6F and heterologous L5C–L5F and L6C–L6F treatments; all *P*<0.0001). However, no significant differences were observed between homologous and heterologous treatments within the same developmental stage (L5: *P*=0.9; L6: *P*=0.9; Table S10; Fig. 5A). Estimated marginal means showed that TAC varied primarily according to developmental-stage combination rather than treatment (Tables S10 and S11). The highest TAC values were observed in the heterologous L5C–L6F and the homologous L5F–L6F mixed developmental-stage groups, whereas the lowest values occurred in L6 offspring (L6F–L6F and L6C–L6F). The heterologous L6C–L5F mixed group and the L5 stage group (L5F–L5F and L5C–L5F) showed intermediate values between L6 (L6F–L6F and L6C–L6F) and the mixed-stage groups (L5C–L6F and L5F–L6F). Although the GAMLSS detected a small but significant effect of treatment on the fitted mean response (Table S11), homologous and heterologous treatments had nearly identical estimated marginal means within each developmental-stage combination and were not consistently separated by the Sidak-adjusted comparisons (Table S12). Overall, these results indicate that the developmental stage, particularly the sequence of parent priming and offspring challenge, explained most of the variation in TAC, whereas treatment identity contributed comparatively little to the observed response (Fig. S2A).

For CAT content, the AIC showed that the best model for this parameter was the gamma GAMLSS with the dispersion parameter (*σ*) dependent on the treatment (m_1_: AIC=1815.74), compared to constant dispersion (m_0_: AIC=1816.51) or dispersion dependent on the developmental stage (m_2_: AIC=1817.08). The model showed that the developmental stage significantly affected CAT activity. In general, the mean CAT was lower in L5 than in L6 (β=0.65±0.19, *P*=0.001). The treatment did not affect either the mean or dispersion of CAT activity (Table S13). Pairwise comparisons supported higher CAT activity in L6 than in L5 within each treatment; however, between stage and treatment, not all contrasts were significant. For example, control L5C–L5C did not differ from heterologous L6C–L6F or homologous L6F–L6F (*P*>0.27), but its CAT content was significantly lower than that of control L6C–L6C (*P*=0.01). Homologous L5F–L5F differed only from homologous L6F–L6F (*P*=0.01), but no differences were found between control L6C–L6C, heterologous L6C–L6F, and homologous L5F–L5F (*P*>0.23). Within each developmental stage, no significant differences were detected among treatments (all *P*>0.65; Table S14; Fig. 5B). Overall, these results indicate that CAT activity increases with the developmental stage. To further evaluate immune responses in the mixed developmental-stage treatments, estimated marginal means from the CAT activity model were compared across all developmental-stage and treatment combinations (Tables S15 and S16). Catalase activity increased markedly in the mixed developmental-stage groups (L6C–L5F, L5C–L6F and L5F–L6F) relative to L5 (L5F–L5F and L5C–L5F) and L6 (L6F–L6F and L6C–L6F), with the highest estimated mean observed in the heterologous L6C–L5F treatment, although this group exhibited substantial variability and overlapped statistically with several other groups. The homologous L5F–L6F treatment showed the highest statistically distinct CAT activity, whereas the heterologous L5C–L5F treatment displayed the lowest values. The GAMLSS detected significant effects of both developmental stage and treatment on the mean response (Table S15), with homologous treatments exhibiting higher fitted CAT activity than heterologous treatments. Nevertheless, pairwise comparisons indicated considerable overlap among several homologous and heterologous groups, suggesting that developmental-stage combination had a stronger influence on CAT activity than treatment identity alone (Fig. S2B).

For SOD activity, based on AIC, the most parsimonious model was the gamma GAMLSS with the dispersion parameter (*σ*) dependent on the developmental stage (m_2_: AIC=899.22), compared to constant dispersion (m_0_: AIC=1008.15) or dispersion dependent on treatment (m_1_: AIC=984.3). The model showed that the developmental stage had a significant effect on both the mean and the dispersion of SOD activity. Overall, SOD activity was higher in L5 than in L6 (β=−0.33±0.05, *P*<0.0001), and variability was greater in L5 compared to L6 (σ: t=12.65, *P*<0.0001; Table S17). Pairwise comparison within the L5 stage indicated that control L5C–L5F larvae showed higher SOD activity than homologous L5F–L5F larvae (*P*=0.03). The differences between control L5C–L5C and heterologous L5C–L5F (*P*=0.7), and between homologous L5F–L5F and heterologous L5C–L5F (*P*=0.97), were not significant. A similar pattern was observed in the L6 stage, where the only significant difference occurred between control L6C–L6C and homologous L6F–L6F larvae (*P*=0.03), with control larvae having higher activity. Across developmental stages, SOD activity was generally higher in L5 than in L6 for all treatments (control, heterologous, and homologous, *P*<0.001). When examining specific stage and treatment combinations, control L5C–L5C also showed higher activity than heterologous L6C–L6F (*P*=0.0001) and homologous L6F–L6F (*P*<0.0001). Heterologous L5C–L5F had higher activity than homologous L6F–L6F (*P*=0.003) but not compared to control L6C–L6C (*P*=0.23). Homologous L5F–L5F larvae did not differ from control L6C–L6C (*P*=0.53) or heterologous L6C–L6F (*P*=0.19; Table S18; Fig. 5C). Overall, these results indicate that the developmental stage is also influencing SOD activity. To further characterize SOD activity in the mixed developmental-stage treatments, estimated marginal means were compared across all developmental-stage and treatment combinations (Tables S19 and S20). SOD activity varied primarily with developmental-stage combination, with the lowest values observed in L6 (L6C–L6F and L6F–L6F), intermediate values in L5 (L5C–L5F and L5F–L5F), and the highest activity in all mixed developmental-stage groups (L5C–L6F, L5F–L6F and L6C–L5F). The heterologous L5C–L6F and L6C–L5F treatments exhibited the highest estimated means, although these groups overlapped statistically with their homologous counterparts (L5F–L6F). Consistent with the GAMLSS results, the developmental stage had a significant effect on the mean response, whereas treatment identity did not significantly influence SOD activity (Table S19). Accordingly, homologous and heterologous treatments showed similar SOD activity within each developmental-stage combination, indicating that variation in SOD activity was driven primarily by the developmental stage rather than by treatment identity (Fig. S2C).

Finally, for H_2_O_2_ content, based on the AIC, the best model for this parameter was the gamma GAMLSS with the dispersion parameter (*σ*) dependent on the treatment (m_1_: AIC=1642.51), compared to constant dispersion (m_0_: AIC=1658.69) or dispersion dependent on the developmental stage (m_2_: AIC=1648.07). The model showed that the developmental stage had a significant effect on the mean of hydrogen peroxide levels, with L6 larvae exhibiting higher H_2_O_2_ concentrations than L5 across all treatments (β=1.48±0.2, *P*<0.0001). Additionally, treatment influenced the dispersion of H_2_O_2_, with the homologous treatment showing significantly lower variability compared to the intercept (σ=−0.53±0.155, *P*=0.001; Table S21). Pairwise comparisons indicated that L6-stage larvae consistently showed higher H_2_O_2_ levels than their L5 counterparts. Control L5C–L5C differed significantly from control L6C–L6C (*P*<0.0001), homologous L5–L5F differed from homologous L6F–L6F (*P*<0.0001), and heterologous L5C–L5F differed from heterologous L6C–L6F (*P*<0.0001). Within a given developmental stage, no significant differences were detected between treatments, indicating that H_2_O_2_ levels were relatively uniform among control, homologous, and heterologous larvae of the same stage (L5: *P*>0.25; L6: *P*>0.28). Between stage and treatment, heterologous L5C–L5F and homologous L5F–L5F larvae differed significantly from all L6 treatments (*P*≤0.006). In contrast, control L5C–L5C and homologous L6F–L6F showed similar H_2_O_2_ levels (*P*=0.28; Table S22; Fig. 5D). Overall, these results indicate that the developmental stage affects the H_2_O_2_ activity. To further characterize H_2_O_2_ production in the mixed developmental-stage treatments, estimated marginal means were compared across all developmental-stage and treatment combinations (Tables S23 and S24). The H_2_O_2_ concentration varied primarily with the developmental stage, with the highest levels observed in L6 (L6C–L6F and L6F–L6F) offspring, intermediate levels in L5 (L5C–L5F and L5F–L5F), and the lowest concentrations in all mixed developmental-stage groups (L5C–L6F, L5F–L6F and L6C–L5F), which did not differ significantly from one another. Although the GAMLSS detected a small but significant effect of treatment on the fitted mean response (Table S23), homologous and heterologous treatments showed similar estimated marginal means within each developmental-stage combination and were not separated by the Sidak-adjusted pairwise comparisons (Table S24). These findings indicate that developmental stage was the primary determinant of H_2_O_2_ production, whereas treatment identity contributed comparatively little to the observed variation (Fig. S2D).

## DISCUSSION

This study examined the effect of developmental stage on the costs and benefits of TGIP in *S. frugiperda*. The primed group had marginally significant differences in survival compared with the control group not challenged. However, the control group survived better than the heterologous challenges and the homologous challenge in L6 (Figs 1 and 2). We found evidence for immune priming in the L5 stage but not in L6. The immune response and oxidative stress were higher in L6 than in L5, independently of the immune treatment. Below, we discuss these findings, considering previously described costs of immune activation, the importance of verifying infection clearance before the second challenge, and the developmental constraints that may underlie immune priming.

Regarding immune activation, our results indicated that the LD25 dose does not produce an apparent survival cost in the parental generation. The lack of differences in survival in primed parents is important in ruling out the possibility that differences observed upon subsequent challenge arise from selective mortality during the priming phase (Rutkowski et al., 2023). However, another meta-analysis by Nystrand and Dowling (2020) proposes that although immune responses do not always impose immediate or detectable costs, the overall pattern across metazoans is that immune challenges tend to reduce survival and reproduction in a strongly context-dependent manner. They suggested that the cost of immune activation may depend on factors such as dose, life stage, or experimental context. Similarly, Ardia et al. (2012) found that immune activation increased metabolic rate in insects, indicating an internal energetic cost that did not necessarily translate into reduced antimicrobial activity or decreased survival. Experimental studies also report that immune activation produces physiological responses without clear developmental costs. For example, in *Drosophila melanogaster*, Leitão et al. (2024) showed that immune stimulation with a wasp homogenate induced melanization, hemocyte proliferation, and metabolic alterations, yet these responses did not affect developmental time. Here, we show that the developmental stage at which parents are primed is associated with the potential costs of TGIP. Specifically, parental priming at the L6 stage imposed costs on offspring. These findings raise the possibility that individuals approaching pupation (L6), compared with those at an earlier larval stage (L5), allocate resources to completing development rather than to immune investment in either themselves or their offspring. Such a shift in resource allocation would be consistent with life-history trade-offs between development and immunity. Transcriptomic analyses further revealed stage-specific differences in immune gene expression, with several immune-related genes showing higher expression in fifth-instar larvae than in sixth-instar larvae (Wang et al., 2025). However, this interpretation remains speculative and warrants further experimental investigation.

Concerning the offspring, homologous L5F–L5F larvae developed faster than control L5C–L5C larvae (Fig. 3). Although heterologous L5C–L5F larvae showed a lower mean developmental time than the control, this difference was not statistically significant. Notably, L5C–L5F larvae nonetheless spent more time in the larval stage than L5F–L6F or L5C–L6F larvae. As occurs in immune priming within generations, we suggest that TGIP can either accelerate or delay development depending on context (Carmona-Peña et al., 2022). This interpretation is further supported by the absence of developmental-time differences between L6C–L6F, L6C–L5F, L6F–L6F, L6F–L5F, and the control L6C–L6C. According to the bet-hedging hypothesis, some organisms accelerate development to escape infection during metamorphosis, and this strategy has been documented in insects subjected to immune priming within generations (Beaumont et al., 2009; Menu et al., 2010; Carmona-Peña et al., 2022). However, in species such as *Tenebrio molitor*, individuals with immune priming within generations exhibit prolonged larval development (Contreras-Garduño et al., 2019). This distinction is important because accelerated development often yields smaller adult body size and reduced fitness (Metcalfe and Monaghan, 2001; Roth et al., 2010), and a delay in development may negatively impact reproduction (Contreras-Garduño et al., 2019). Thus, the timing of priming and challenge relative to larval stage influences developmental rate, an aspect that should be considered in future studies when reconciling apparently conflicting results when testing the impact of immune priming (within and across generations) on the developmental rate.

Our results corroborated TGIP in *S. frugiperda* and demonstrated that it depends on the developmental stage in which the offspring is challenged. TGIP was demonstrated in terms of survival at L5 (Fig. 1): the control group (non-challenged) and the homologous group at L5 did not differ in survival, but the heterologous groups survived for fewer days than the control group. Hence, our results support previous studies that provided evidence for TGIP (Little et al., 2003; Moret, 2006; Sadd and Schmid-Hempel, 2006; Roth et al., 2010; Tate et al., 2017). A recent review suggests that survival within generations is a key parameter to test the immune priming phenomenon in invertebrates because of the difficulty to find key immune effectors that may explain the immune priming (not all parameters were related to immune priming; Lanz-Mendoza et al., 2024), and our results support this because we find evidence for TGIP at the survival level but not at the mechanistic level (see below). Our results also revealed a key factor for the immune priming outcome: the developmental stage.

Notably, in *Spodoptera*, TGIP has been tested only in single instars: Wilson and Graham (2015) tested it in L3 and Wilson et al. (2021) in L4. By focusing on a single stage, important information about plasticity across development may be missed (Tetreau et al., 2019). Here, TGIP was found in L5, but not in L6 (Figs 1 and 2). Indeed, the homologous and heterologous groups challenged at L6 survived fewer days than the control group. We suggest that L5 and L6 pay different costs for immune priming. Immune capacity is known to decline with age in several insects, including mosquitoes and mealworms (Hillyer et al., 2005; Amaro-Sánchez et al., 2023). During the larval phase, insects progressively accumulate nutrient reserves in the fat body (Arrese and Soulages, 2010). However, during the final larval instar, larvae undergo endocrine reprogramming, characterized by a decline in juvenile hormone and pulses of 20-hydroxyecdysone, that prepares tissues for metamorphosis (Jindra et al., 2013; Rantala et al., 2020; Gao et al., 2022). Thus, the last larval instar functions as a critical energetic provisioning phase before pupation (Gao et al., 2023; Arrese and Soulages, 2010). Because metamorphosis imposes substantial metabolic and physiological demands, including increased mitochondrial activity, elevated energy expenditure, and vulnerability to pathogens (Merkey et al., 2011; Rolff et al., 2019), larvae may not be able to simultaneously meet these demands and mount an immune priming response. This likely explains the absence of TGIP in L6. Consistently, in the scallop *Chlamys farreri* and the moth *Manduca sexta*, the effectiveness of immune priming was also associated with the developmental stage (Yue et al., 2013; Trauer and Hilker, 2013). Overall, these results support that the developmental stage at which immune challenges occur determines whether TGIP is present or absent in *Spodoptera* in terms of survival.

Although survival patterns differ strongly between stages, the immune parameters (PO, lytic activity) did not show significant differences between homologous and heterologous treatments (Fig. 4). Even though there was a higher immune response in the L6 stage compared with the control group, there were no differences between homologous and heterologous groups. These findings reinforce that single immune parameters are poor predictors of immune memory (Lanz-Mendoza and Contreras-Garduño, 2022). In *M. sexta*, for example, the expression of TGIP depended on the specific immune gene evaluated (Trauer and Hilker, 2013; Trauer-Kizilelma and Hilker, 2015). Therefore, the parameters measured here likely capture only a fraction of the complexity underlying TGIP, and additional components, such as hemocytes, antimicrobial peptides, or Toll-pathway activity, may provide a better mechanistic understanding. We suggest these specific measurements because Toll signaling promotes antimicrobial peptide production, is induced by fungal and Gram (+) bacterial infections, and is mediated by hemocyte components (Nappi and Vass, 1998; Lemaitre and Hoffmann, 2007; Mylonakis et al., 2016). For example, in *Spodoptera exigua*, infection with *Beauveria bassiana* induced the expression of attacin, cecropin, gallerimycin, gloverin, hemolin, and transferrin (Park and Kim, 2012). Future experiments incorporating immune gene expression analyses could help identify potential effector responses that distinguish homologous from heterologous groups.

Nevertheless, while survival differences indicate that TGIP can result in enhanced resistance under specific developmental conditions, the mechanisms responsible for this protection remain unresolved. In particular, the immune parameters measured here did not directly assess pathogen control. Quantifying fungal burden after challenge would provide a complementary functional assessment of immune protection by determining whether primed larvae are able to reduce or clear the infection more efficiently. Therefore, studies should integrate survival analyses with measurements of pathogen load to better identify the mechanisms underlying TGIP (Little et al., 2005; Lanz-Mendoza et al., 2024). The heterologous (L5C–L6F and L6C–L5F) and homologous (L5F–L6F) mixed developmental-stage treatments reinforce our interpretation that the developmental stage differs in physiology and that L6 may be more susceptible than L5. Indeed, we were unable to incorporate the homologous mixed group L6F–L5F because most organisms died. Although these comparisons expanded the developmental combinations evaluated, they did not alter the main conclusions of the study. Across the immune and oxidative stress parameters, developmental stage remained the primary source of variation, whereas homologous and heterologous treatments generally produced similar responses within each developmental-stage combination. Moreover, the mixed-stage groups did not exhibit a consistent immune profile that could explain the differences observed in survival. These findings reinforce the conclusion that the developmental stage at which priming and challenge occur is a strong determinant for the TGIP outcome.

Regarding the antioxidant defense, CAT and H_2_O_2_ activities were higher in L6, whereas the TAC and SOD were more strongly activated in L5. The stronger production of H_2_O_2_ in L6 suggests that this stage experiences higher oxidative stress than L5. This is supported because the control group in L6 also had more H_2_O_2_ than the control group in L5 (Fig. 5). During microbial challenge, reactive oxygen and nitrogen species (ROS and RNS, respectively) act to damage pathogen structures (Eleftherianos et al., 2021; Buchon et al., 2014; Carter and Hurd, 2010; Kumar and Klessig, 2003). Nitric oxide produced by phagocytic hemocytes can damage membranes, proteins, and nucleic acids directly or through derived compounds such as peroxynitrite (ONOO^−^) or hydroxyl radicals (•OH) (Nappi et al., 2000; Rivero, 2006). However, excessive or prolonged ROS/RNS production can harm host tissues. In *T. molitor*, activation of the phenoloxidase cascade caused tissue damage indicative of self-harm (Sadd and Siva-Jothy, 2006). This aligns with our finding that L6 larvae, despite mounting stronger immune responses, exhibited lower survival and lacked memory. The intensity of the immune response in L6 may therefore generate oxidative self-damage, prevent the establishment of memory, and, ultimately, reduce survival. Taken together, our results indicate that L5 larvae can mount an immune response robust enough to generate immune priming and controlled enough to avoid high oxidative cost. In contrast, L6 larvae mount a more intense immune response that does not translate into improved survival. Instead, this exacerbated response may overload the organism during a developmentally demanding stage, leading to what resembles immunopathology, autoimmune-like damage, or immune reactivity (Moret, 2003). These findings warrant further investigation, as they appear somewhat counterintuitive given that higher expression of immune-related genes has been reported in fifth-instar larvae than in sixth-instar larvae (Wang et al., 2025). One possible explanation is that immune activation in sixth-instar larvae may impose substantial energetic or physiological costs, potentially depleting resources required for other essential functions and thereby reducing survival. Alternatively, excessive or prolonged immune activation itself may contribute to mortality if the resources required to regulate or terminate the immune response become limiting. However, these possibilities remain speculative and require direct experimental evaluation.

This study provides evidence that TGIP in *S. frugiperda* is strongly dependent on the developmental stage in which it is induced. Our findings align with the hypothesis that stage-specific physiology plays a fundamental role in shaping costs and benefits (Tetreau et al., 2019). This plasticity may contribute to the ecological success of *S. frugiperda*, a major agricultural pest known for its resilience and rapid adaptation (Wan et al., 2021). Understanding the mechanisms behind stage-specific immune priming will improve our ability to predict host–pathogen interactions and may have implications for pest management strategies.

## MATERIALS AND METHODS

### Insect rearing conditions

*S. frugiperda* larvae were obtained from a laboratory colony. Fifth- and sixth-instar larvae (L5 and L6) were used for the experiments. Larvae were reared on an artificial cornmeal-based diet and maintained in an incubator at 25±2°C under a 12:12-h light:dark photoperiod. Upon adult emergence, individuals were maintained in a dark room and provided with a 10% honey solution *ad libitum*. Adults were kept in constant darkness to reflect their nocturnal activity (Goh et al., 1990; de la Luz Sierra-Ruíz et al., 2022).

### Experimental design

#### Fungal strains

Two strains of the entomopathogenic fungus *Metarhizium brunneum* were used: the Cherry strain, which carries a red fluorescent marker, and the GFP strain, which expresses a green fluorescent marker. These markers favor the detection of fungal infections under a fluorescence microscope (Castro-Vargas et al., 2017).

#### Dose determination

To establish the doses used in the experiments, we determined a lethal dose causing 100% mortality (LD100) and a sublethal dose causing approximately 20–30% mortality (hereafter referred to as LD25; Medina-Gómez et al., 2018). Two strains of the entomopathogenic fungus *M. brunneum* were used: the Cherry strain, which carries a red fluorescent marker, and the GFP strain, which expresses a green fluorescent marker. These markers favor the detection of fungal infections under a fluorescence microscope (Castro-Vargas et al., 2017).

A total of 274 larvae from each instar (L5 and L6) were used. For both instars, larvae were divided into four groups: three groups were injected with 1 μl of different concentrations of the Cherry strain of *M. brunneum* diluted in 0.001% Tween 80 (5, 200, or 2000 conidia/μl), and a control group was injected with 1 μl of Tween 80 alone. Injections were administered between the sixth and seventh abdominal segments using a 10 μl microsyringe with a removable needle (Model 701 RN, Hamilton Company, Reno, NV, USA). After injection, larvae were individually maintained in 24-well plates containing the diet. Survival was recorded daily, along with the developmental time between each stage. Based on these, the LD25 dose corresponded to 5 conidia/μl for the GFP strain (priming), while the LD100 dose corresponded to 2000 conidia/μl for the Cherry strain (challenge) (Fig. S3). A total of 274 larvae from each instar (L5 and L6) were used. For both instars, larvae were divided into four groups: three groups were injected with 1 μl of different concentrations of the Cherry strain of *M. brunneum* diluted in 0.001% Tween 80 (5, 200, or 2000 conidia/μl), and a control group was injected with 1 μl of Tween 80 alone. Injections were administered between the sixth and seventh abdominal segments using a 10 μl microsyringe with a removable needle (Model 701 RN, Hamilton Company, Reno, NV, USA). After injection, larvae were individually maintained in 24-well plates containing the diet. Survival was recorded daily, along with the developmental time between each stage. Based on these observations, the LD25 dose corresponded to 5 conidia/μl, while the LD100 dose corresponded to 2000 conidia/μl (Fig. S3).

### TGIP experiment

TGIP was assessed through a two-phase experiment. In the first phase, the immune response of the parental generation (F0) was activated using an LD25 dose of the GFP strain of *M. brunneum*. Two experimental groups of larvae were established: a memory group and a control group (*N*=383). Larvae were inoculated during either the L5 or L6 instar. In the initial immune challenge (priming), individuals in the memory group were injected with the fungal suspension (L5: *n*=95; L6: *n*=96), while those in the control group received 1 μl of 0.001% Tween 80 (L5: *n*=96; L6: *n*=96). Larvae were maintained individually in 24-well plates containing the diet until pupation, after which individuals were sexed and randomly paired within the same treatment group (i.e. memory female×memory male or control female×control male) in plastic containers.

**Fig. 6.**
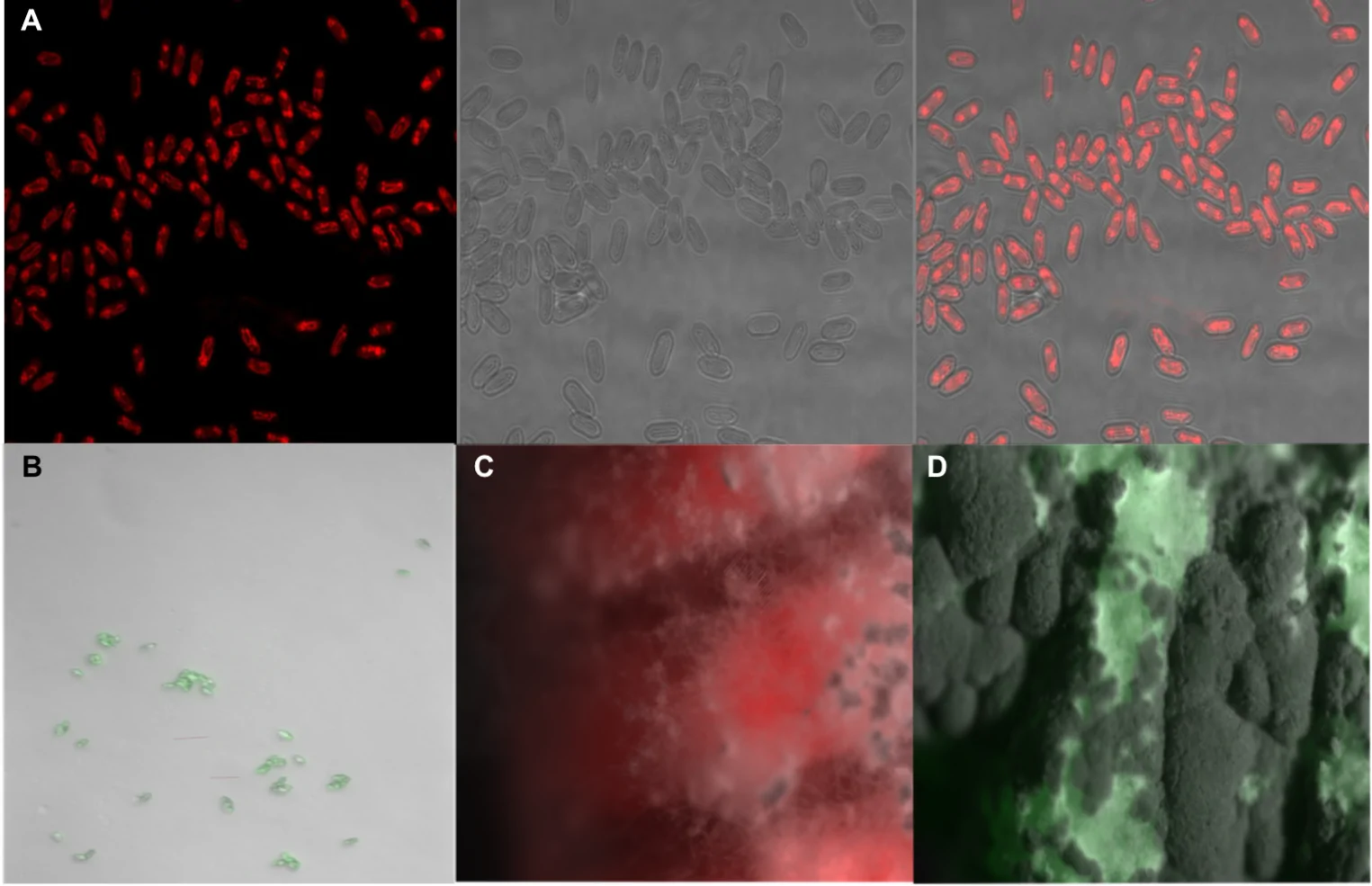
**Corroboration of the absence of infection overlap.** (A–D) Conidia recovered from infected larvae of the homologous group at the L5 or L6 stage were observed under a fluorescence microscope. After the second challenge (A), only Cherry-labeled conidia were detected, indicating the absence of overlap with the first infection with GFP-labeled conidia. In panel B, we show how the GFP conidia look like. Images were captured at 40× magnification under red or green fluorescence to detect Cherry-labeled conidia. Panel C shows offspring larvae infected with the Cherry strain at LD100, whereas panel D shows parental larvae infected with the GFP strain at LD25.

Upon adult emergence, insects were provided with a 10% honey-water solution on cotton and folded wax paper strips for oviposition, which were replaced daily. Egg masses were separated from adults, and upon hatching, offspring were reared on an artificial diet under controlled conditions. Survival and developmental times at each stage were recorded. Using fluorescence microscopy, we confirmed that infections from the first and second challenges did not overlap. Twenty-four hours before the second challenge, no GFP conidia or pieces of conidia derived from parents were detected, and 24 h after the second challenge, only Cherry conidia were observed (Fig. 6). This is an important methodological consideration in immune priming experiments to confirm that the initial infection has been cleared before administering the second exposure. Pathogen persistence could otherwise confound the interpretation of enhanced survival as immune memory. Only a few studies have explicitly verified pathogen clearance before administering a secondary challenge (e.g. Sadd and Schmid-Hempel, 2006; Castro-Vargas et al., 2017; Mondotte et al., 2018).

**Fig. 7.**
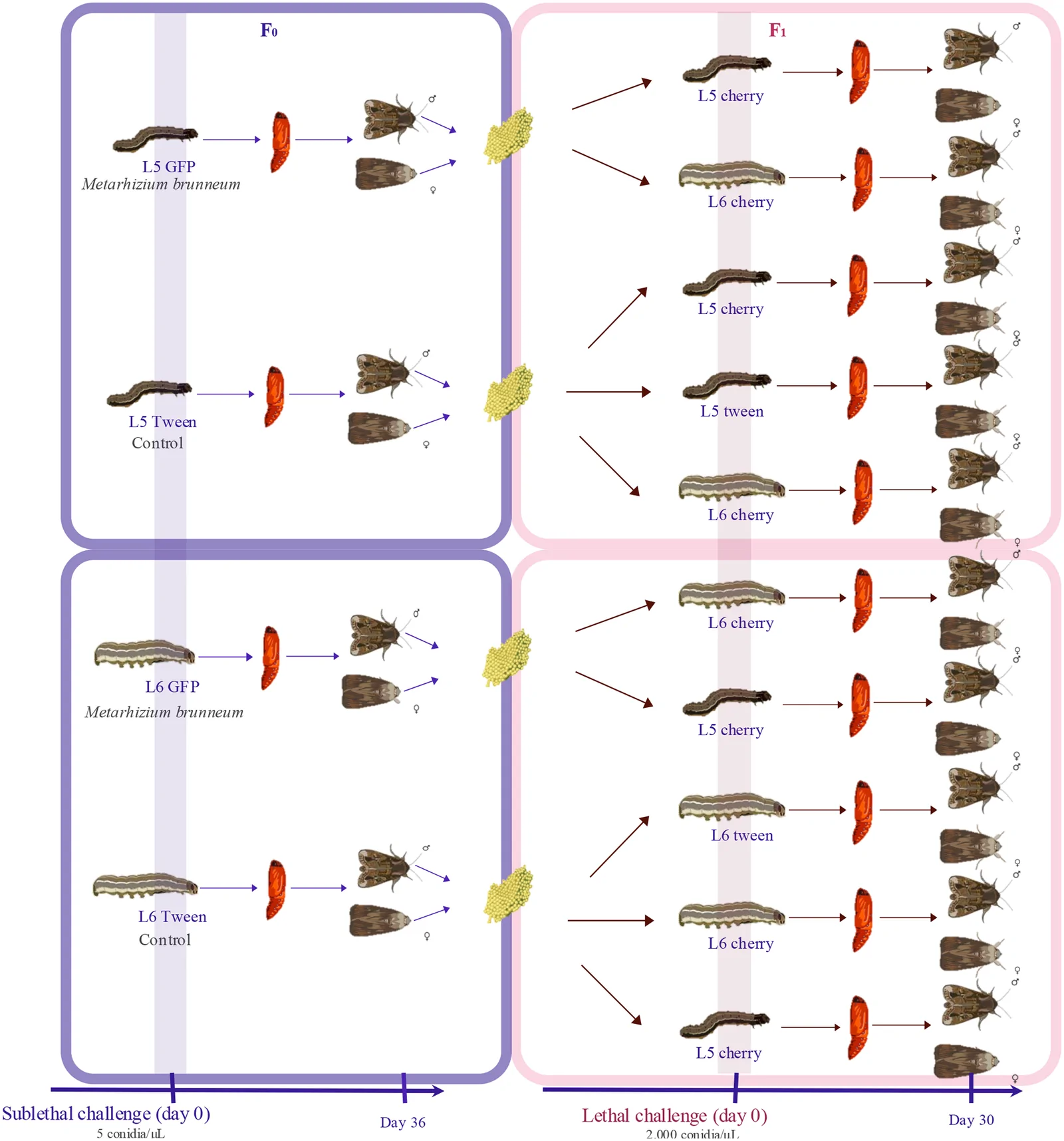
**Experimental design to assess transgenerational immune priming.** The purple box highlights the first phase of the experiment, in which two treatments were applied to parental larvae: experimental (5 conidia/μl) and control (Tween 80), administered at both L5 and L6 stages. The pink box represents the second phase, in which offspring from the control group were divided into three treatments: injected with Tween 80, challenged at L5, or challenged at L6 with 2000 conidia/μl. For homologous groups, offspring were challenged at the same stage as their parents (L5 or L6). Hemolymph was collected 24 h after the second challenge to evaluate immune response and oxidative stress, and survival and duration of each phase were recorded for all groups. Numbers on the *x*-axis indicate the total duration (in days) of the parental (35 days) and offspring (30 days) life cycles throughout the experiment.

For the second phase of the experiment, offspring that survived to the L5 or L6 larval stage from the F0 treatments were injected. Offspring were assigned to the following treatments (total *n*=639), defined by parental treatment (control or fungus), offspring treatment (control or fungus), and larval stage at challenge. Homologous challenge treatments were those in which both parents and offspring were challenged with *M. brunneum*. The control treatment consisted of a 1 µl injection of Tween 80 into both parents and offspring of the same larval stage (L5C–L5C or L6C–L6C). For the larvae with a homologous challenge, treatments were applied at the same larval stage, L5F–L5F (*n*=77) and L6F–L6F (*n*=148), where parents were challenged with an LD25 dose and offspring with an LD100 dose (2000 conidia/μl, *M. brunneum,* Cherry strain) at L5 and L6 stages, respectively. Heterologous challenge treatments were those in which parents received a control injection (Tween 80) and offspring were challenged with the fungus at the same larval stage, including L5C–L5F (*n*=44) and L6C–L6F (*n*=22). To assess the effect of the larval stage at challenge, additional treatments involved challenges applied at different larval stages between parents and offspring. These included homologous challenge with different stages, L5F–L6F (*n*=56) and L6F–L5F (*n*=12), in which parents were challenged with an LD25 dose at one larval stage and offspring were challenged with an LD100 dose at the opposite stage. Finally, heterologous challenge with different stages included L5C–L6F (*n*=25) and L6C–L5F (*n*=8), where parents received a control injection and offspring were challenged with the fungus at a different larval stage (Fig. 7).

### Hemolymph extraction

After 24 h of injection, 185 individuals were separated, and 30 μl of hemolymph was extracted from each larva. The hemolymph was diluted in 200 μl of PBS (Sigma-Aldrich), and the samples were stored in a freezer at −80°C. It is important to note that these individuals were excluded from survival and development analyses. The following immune response measures were analyzed from these samples across the different treatments: lytic activity, phenoloxidase activity, a prooxidant, and various antioxidants. Protein levels in the hemolymph were quantified to ensure that any significant differences in immune response were not due to varying initial protein quantities across groups (see Results). To analyze the costs during development, the time (in days) taken by each larva to reach the pupal stage was recorded, followed by the time spent in the pupal stage and the adult stage. Survival after each challenge was also recorded to assess the benefits of immune priming (Fig. 1). To further evaluate the influence of developmental stage on immune responses, additional analyses were performed using the mixed developmental-stage treatments (L5C–L6F, L5F–L6F and L6C–L5F). These treatments were originally established to assess survival following priming and challenge. Because survival after the second challenge was reduced in the mixed-stage groups, fewer individuals remained available for hemolymph extraction, resulting in smaller sample sizes for the immune assays compared with the main experimental groups. Therefore, immune parameters for the mixed-stage treatments were analyzed using all available samples, and these results are presented in Figs S1 and S2.

### Immune and antioxidant assays

#### Phenoloxidase (PO) activity assay

We dissolved 0.004 g of L-3,4(OH)_2_-phenylalanine (L-DOPA; Sigma-Aldrich) in 1 ml of PBS. The solution was covered with aluminum foil and agitated for 20 min until fully dissolved. In a 96-well ELISA plate, two wells were used as controls, receiving 90 μl of PBS and 10 μl of L-DOPA. The remaining wells were treated similarly, with 10 μl of L-DOPA, 40 μl of PBS, and 50 μl of the sample (*n*=184). After 15 min of incubation at room temperature, the absorbance of each well was measured at 490 nm using a Multiskan GO microplate reader (Thermo Fisher Scientific) every 3 min for 30 min. To determine PO activity, the slope of the linear regression curve for each sample's reaction was calculated. The maximum rate of change in absorbance (*V*_max_) was used for subsequent analyses and expressed as ΔAbs/min.

#### Lytic activity assay

Lytic activity was measured using a *Micrococcus lysodeikticus* lysis assay. A reagent solution was prepared by dissolving 0.072 g of *M. lysodeikticus* (Sigma-Aldrich) in 19.2 ml of PBS. Two wells in the ELISA plate were used as controls, receiving only 230 μl of PBS. The remaining wells were filled with 200 μl of *M. lysodeikticus* and 30 μl of the sample (*n*=185). The plate was incubated for 15 min at room temperature. Absorbance was measured at 490 nm following the same incubation time and reading protocol described for the phenoloxidase assay. Lytic activity was calculated as the slope of the linear regression between absorbance and the degradation time of *M. lysodeikticus*. The maximum rate of change in absorbance (*V*_max_) was used for subsequent analyses and expressed as ΔAbs/min.

#### TAC quantification

A TAC kit (catalog #K274-100; BioVision) was used for the assay. Six wells of an ELISA plate were used to create the Trolox standard curve as follows: 0, 4, 8, 12, 16, and 20 μl of the Trolox standard solution (1 μmol) and 100, 96, 92, 88, 84, and 80 μl of ultrapure water per well. For the remaining wells, 25 μl of protein mask, 25 μl of the sample (*n*=103), and 50 μl of the copper working solution were added. The working solution was prepared by diluting the Cu reagent into the assay solution at a 1:49 ratio. The plate was incubated, covered with aluminum foil, at room temperature for 1.5 h. Absorbance was measured at 570 nm using the Multiskan GO microplate reader (Thermo Fisher Scientific). The total antioxidant amount (nmol) for each sample was determined using the regression curve of the equation, and catalase activity is shown as nmol/H_2_O_2_.

#### Catalase (CAT) activity assay

To determine CAT (nmol), a kit (catalog #A22180; Invitrogen) was used, and the following solutions were prepared: for the Amplex Red solution, 100 μl of DMSO was added to a vial of the Amplex Red reagent; the 1× working solution was prepared by adding 4 ml of hydrogen peroxide (H_2_O_2_) to 16 ml of distilled water; the horseradish peroxidase (HRP) solution was prepared by dissolving the vial of HRP reagent in 200 μl of the 1× working solution; the H_2_O_2_ solution was prepared by diluting 23 μl of the H_2_O_2_ vial in 977 μl of distilled water; and the CAT solution was prepared by dissolving the CAT vial in 100 μl of distilled water. Six wells of the ELISA plate were used to generate the CAT standard curve. One well was used as a blank containing 25 μl of the 1× working solution. The remaining wells were prepared by mixing the catalase solution with the 1× working solution to a final volume of 25 μl as follows: 6.25 μl of 1 U/ml catalase+18.75 μl of 1× working solution; 12.5 μl of 1 U/ml catalase+12.5 μl of 1× working solution; 2.5 μl of 10 U/ml catalase+22.5 μl of 1× working solution; 5 μl of 10 U/ml catalase+20 μl of 1× working solution; and 10 μl of 10 U/ml catalase+15 μl of 1× working solution. For the first phase of the reaction, 12.5 μl of the sample (*n*=118) and 12.5 μl of the 40 μM H_2_O_2_ solution were added to the remaining wells. The plate was covered with aluminum foil and incubated for 30 min at room temperature. To prepare the 100 μM Amplex Red solution, 50 μl of the Amplex Red solution and 20 μl of the HRP solution were diluted in 4.93 ml of the 1× working solution. 25 μl of the 100 μM Amplex Red solution was then added to the wells containing the samples. The plate was covered again, incubated at 37°C for 30 min, and absorbance was measured at 560 nm using the Multiskan GO microplate reader (Thermo Fisher Scientific). The CAT content (nmol) for each sample was determined using the regression curve equation.

#### SOD assay

A kit (catalog #19160-1KT-F; Sigma-Aldrich) was used to determine SOD activity. For the working solution, 1 ml of Water-Soluble Tetrazolium (WST) solution from the kit was diluted in 19 ml of buffer solution. The enzyme was centrifuged for 5 s, and 15 μl was diluted in 2.5 ml of buffer solution to prepare the enzyme solution. Three wells of an ELISA plate were used as blanks and prepared as follows: in the first blank well, 10 μl of ultrapure water, 100 μl of the working solution, and 10 μl of the enzyme solution were added; in the second blank well, 10 μl of the sample, 100 μl of the working solution, and 10 μl of the buffer solution were added; and in the third blank well, 10 μl of ultrapure water, 100 μl of the working solution, and 10 μl of the buffer solution were added. The remaining wells were filled with 10 μl of the sample (*n*=116), 10 μl of the enzyme solution, and 100 μl of the working solution. The plate was covered with aluminum foil and incubated at 37°C for 20 min. Absorbance was measured at 450 nm using the Multiskan GO microplate reader (Thermo Fisher Scientific). SOD activity was calculated using the following formula: SOD activity (inhibition rate%)=[(AB1−AB3)−(Asample−AB2)]/(AB1−AB3)×100%, where AB1=absorbance of blank 1; AB2=absorbance of blank 2; AB3=absorbance of blank 3; sample=absorbance of the sample.

### Hydrogen peroxide (H_2_O_2_) quantification

The amount of H_2_O_2_ was determined using a kit (catalog #A22188; Thermo Fisher Scientific). The following standard solutions were prepared: for the 10 mM Amplex Red solution, the Amplex Red vial was dissolved in 60 μl of DMSO; for the 1× solution, 4 ml of the 5× solution was diluted in 16 ml of deionized water; to prepare a 10 U/ml HRP solution, the HRP vial was dissolved in 1 ml of the 1× solution; and a 20 mM H_2_O_2_ solution was prepared by diluting 22.7 μl of the 3% H_2_O_2_ vial in 977 μl of the 1× solution. Eight wells of an ELISA plate were used to create the H_2_O_2_ standard curve. Different volumes of the 10 nM H_2_O_2_ solution and 1× buffer were added to each well to achieve a total volume of 50 μl per well, corresponding to the following concentrations of H_2_O_2_: 10, 8, 6, 4, 2, and 0 nM. Additionally, a solution containing 100 μM Amplex Red and 0.2 U/ml of HRP was prepared by diluting 50 μl of the 10 mM Amplex Red solution and 100 μl of the HRP solution into 4.85 ml of the 1× solution. The remaining wells were filled with 25 μl of the sample (*n*=116) and 25 μl of the 100 μM Amplex Red and 0.2 U/ml of the HRP solution. The plate was covered with aluminum foil and incubated for 30 min at room temperature. Absorbance was measured at 560 nm using the Multiskan GO microplate reader (Thermo Fisher Scientific), and the amount of H_2_O_2_ (nmol) in each sample was calculated using the linear regression equation.

### Statistical analysis

To analyze survival, the Kaplan–Meier log-rank test was performed. The number of larvae hatched from parents and the survival percentage of offspring by treatment were compared using analysis of variance, followed by a Tukey post-hoc test. These analyses were conducted using the IBM SPSS statistical software. To compare the number of days spent by individuals in the larval and pupal stages across different treatments, the Shapiro–Wilk normality test was first performed, revealing that the data were not normally distributed. For each immune response and antioxidant response measurement, three GAMLSSs were fitted using a gamma distribution. All models included the fixed effects of the developmental stage (L5 or L6) and treatment (heterologous or homologous). The analysis focused on differences in the mean and dispersion parameters among treatments and developmental stages. Absolute values of activity were not reported; instead, statistical comparisons were based on model-derived estimates and pairwise tests. They differ in the dispersion parameter (*σ*) and were modeled in three ways: (1) as a constant, (2) dependent on treatment, or (3) dependent on developmental stage. The most parsimonious model was selected based on the AIC. Model fit was assessed using simulation-based diagnostics with the DHARMa package, verifying that the residuals met assumptions of uniformity, dispersion, absence of outliers, and lack of collinearity with no significant deviations. Finally, marginal mean comparisons were performed using the emmeans package to assess differences between treatments and stages. The analysis was performed in RStudio (version 4.4.1). The data are provided as Datasets 1–3.

## Supplementary Material



10.1242/biolopen.062421_sup1 Supplementary information

Dataset 1.

Dataset 2.

Dataset 3.
