# Supplementary material for: Is the developmental stage important for the plasticity of transgenerational immune priming?
Source: Biol Open. 2026 Aug 27;15(8):bio062421. doi: 10.1242/bio.062421 (PMC13580588; doi:10.1242/bio.062421)
Supplement: Supplementary file 1 [file biolopen-15-062421-s1.pdf]

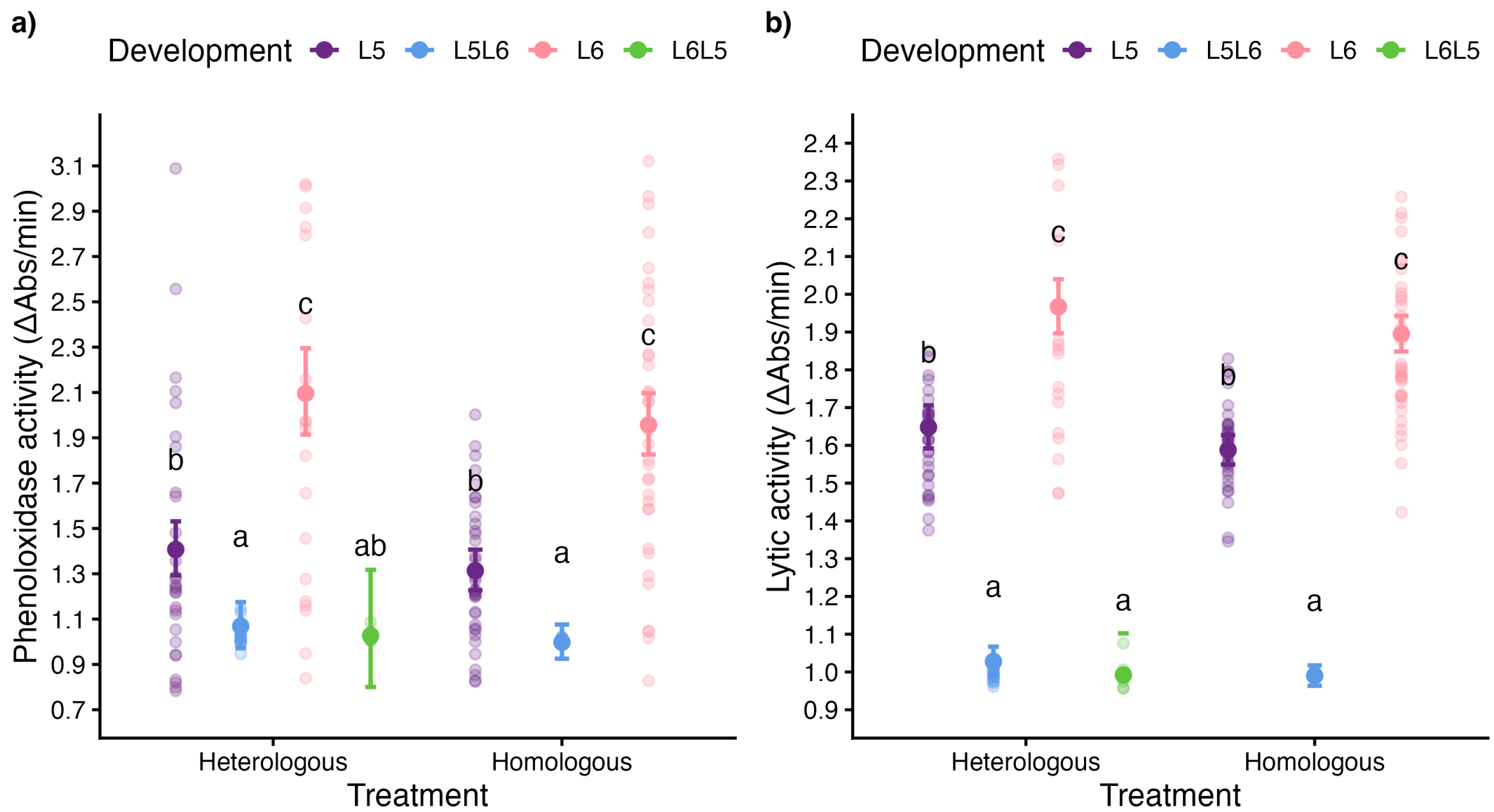

Fig. S1. Predicted immune response parameters across developmental stages and immune challenge treatments. a) Phenoloxidase activity and b) lytic activity. Points represent model-predicted means, and whiskers indicate 95% confidence intervals. Jittered points represent individual biological replicates. Colors denote the developmental-stage group (L5, L6, L5L6, and L6L5), while treatments are shown on the x-axis (homologous and heterologous). Statistical analyses were performed using Gamma generalized additive models for location, scale, and shape (GAMLSS). Pairwise comparisons were conducted using estimated marginal means with Tukey-adjusted P-values. Different letters indicate significant differences among treatment × developmental-stage combinations ($P < 0.05$). Sample sizes were total n = 171: L5 heterologous (L5C–L5F, n = 31), L5 homologous (L5F–L5F, n = 36), L6 heterologous (L6C–L6F, n = 22), L6 homologous (L6F–L6F, n = 36), mixed developmental-stage heterologous (L5C–L6F, n = 20), mixed developmental-stage homologous (L5F–L6F, n = 20), and mixed developmental-stage heterologous (L6C–L5F, n = 6).

Biology Open • Supplementary information

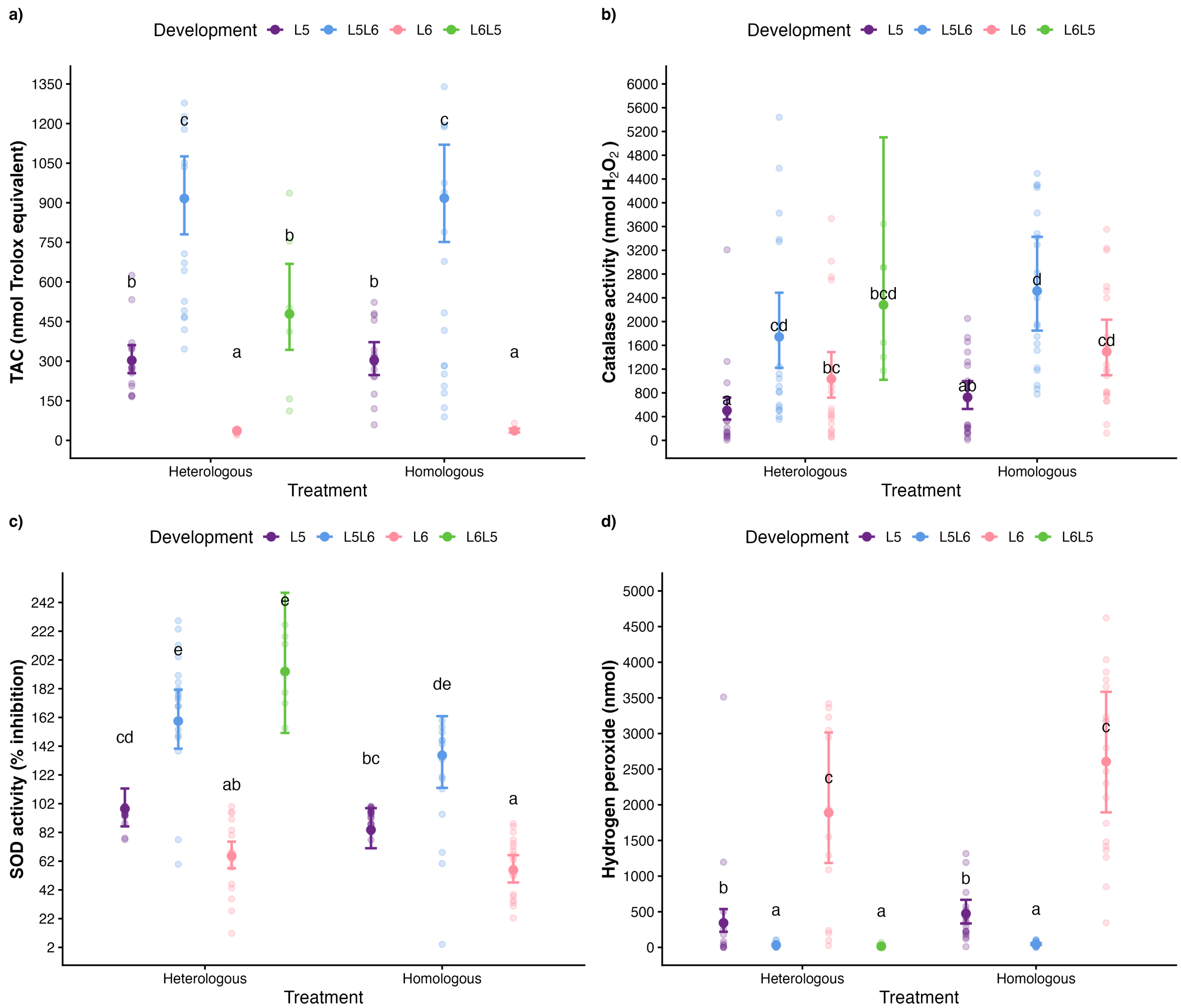

Fig. S2. Predicted antioxidant defense and oxidative status across developmental stages and immune challenge treatments. a) Total antioxidant capacity (TAC), b) catalase activity, c) superoxide dismutase activity (SOD), and d) hydrogen peroxide content (H_2O_2). Points represent model-predicted means, and whiskers indicate 95% confidence intervals. Jittered points represent individual biological replicates. Colors denote the developmental-stage group (L5, L6, L5L6, and L6L5), while treatments are shown on the x-axis (homologous and heterologous). Statistical analyses were performed using generalized additive models for location, scale, and shape (GAMLSS) with a Gamma error distribution. Pairwise comparisons were conducted using estimated marginal means with Tukey-adjusted P-values. Different letters indicate significant differences among treatment × developmental-stage combinations ($P < 0.05$). Sample sizes were total n = 171: L5 heterologous (L5C–L5F, n = 31), L5 homologous (L5F–L5F, n = 36), L6 heterologous (L6C–L6F, n = 22), L6 homologous (L6F–L6F, n = 36), mixed developmental-stage heterologous (L5C–L6F, n = 20), mixed developmental-stage homologous (L5F–L6F, n = 20), and mixed developmental-stage heterologous (L6C–L5F, n = 6).

Biology Open • Supplementary information

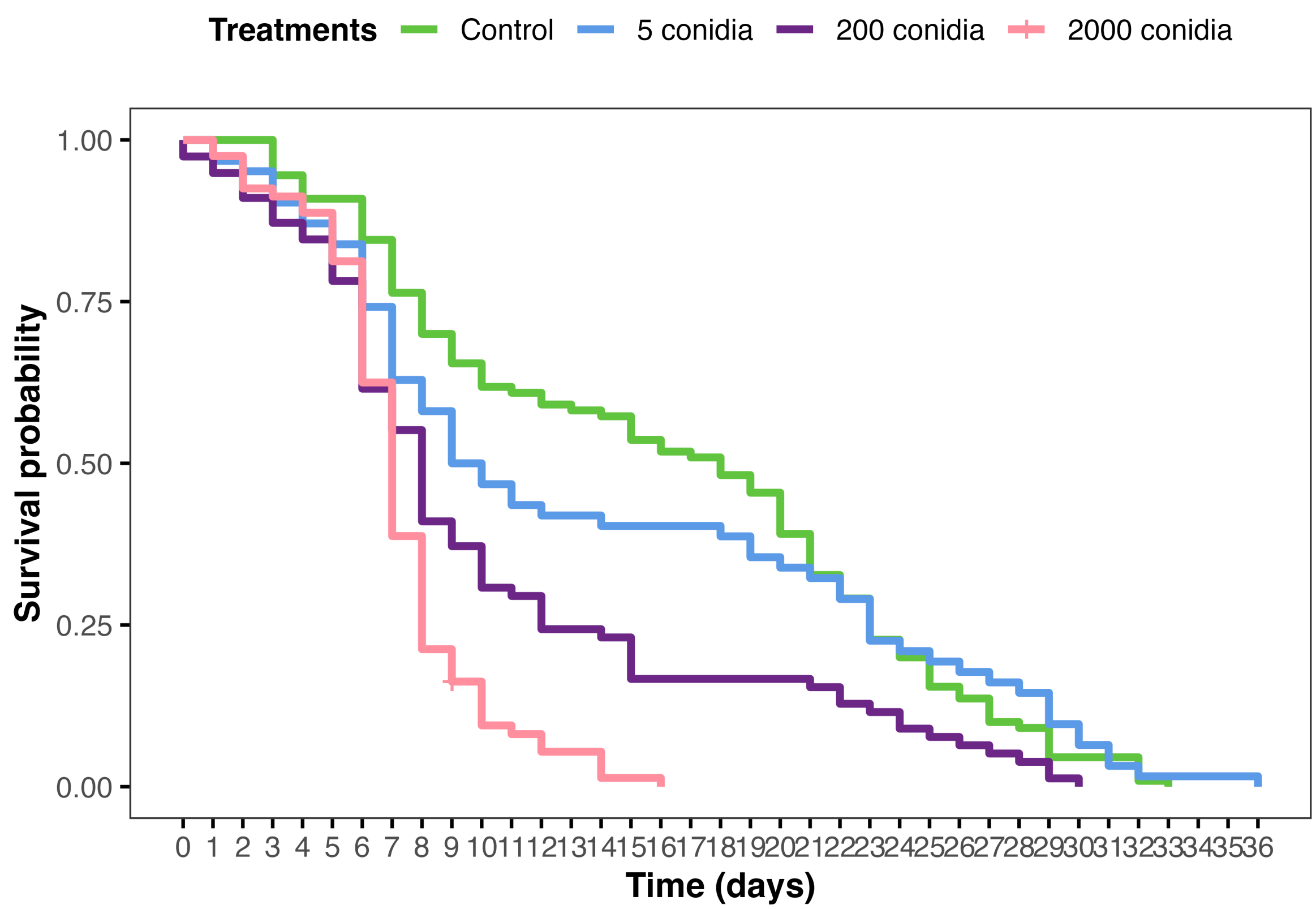

Fig. S3. Kaplan–Meier survival curves of Tenebrio molitor larvae challenged with different concentrations of Metarhizium brunneum conidia (5, 200, and 2,000 conidia/µL) and the Tween control. The lowest dose (5 conidia/µL) did not differ significantly from the control (Log-rank test, $p = 1.00$). In contrast, survival was significantly reduced in larvae challenged with 200 conidia/µL compared with the control ($p = 0.00026$), and the highest dose (2,000 conidia/µL) showed the lowest survival, differing significantly from both the control ($p < 0.0001$) and the 200 conidia/µL treatment ($p = 0.0049$).

Biology Open • Supplementary information

Table S1. Model coefficent for phenoloxidase model. Gamma GAMLSS model as a function of Development (L5 vs L6) and Treatment (Homologous vs Heterologous).

Parameter	Estimate	SE	t-value	p-value	Significance
**Mu (mean)**					
(Intercept)	0.186	0.039	4.768	<0.00001	***
Development (L6)	0.369	0.043	8.622	<0.00001	***
Treatment (Heterologous)	0.180	0.062	2.910	0.004	**
Treatment (Homologous)	0.097	0.047	2.085	0.039	*
**Sigma (dispersion)**					
(Intercept)	-1.357	0.091	-14.860	<2e-16	***
Treatment (Heterologous)	0.382	0.132	2.897	0.004	**
Treatment (Homologous)	0.067	0.123	0.540	0.590	

Table S2. Pairwise comparisons of phenoloxidase values between treatments. Ratios indicate fold-changes of predicted means with 95% confidence intervals; p-values are Tukey-adjusted.

Comparison	ratio	SE	z.ratio	p-value
**Control L5 / Heterologous L5**	**0.836**	**0.052**	**-2.910**	**0.042**
Control L5 / Homologous L5	0.907	0.042	-2.085	0.295
**Control L5 / Control L6**	**0.691**	**0.030**	**-8.622**	**<.0001**
**Control L5 / Heterologous L6**	**0.578**	**0.044**	**-7.213**	**<.0001**
**Control L5 / Homologous L6**	**0.627**	**0.039**	**-7.517**	**<.0001**
Heterologous L5 / Homologous L5	1.086	0.067	1.344	0.760
Heterologous L5 / Control L6	0.827	0.061	-2.557	0.108
**Heterologous L5 / Heterologous L6**	**0.691**	**0.030**	**-8.622**	**<.0001**
**Heterologous L5 / Homologous L6**	**0.751**	**0.055**	**-3.950**	**0.001**
**Homologous L5 / Control L6**	**0.762**	**0.049**	**-4.210**	**0.001**
**Homologous L5 / Heterologous L6**	**0.637**	**0.049**	**-5.885**	**<.0001**
**Homologous L5 / Homologous L6**	**0.691**	**0.030**	**-8.622**	**<.0001**
**Control L6 / Heterologous L6**	**0.836**	**0.052**	**-2.910**	**0.042**
Control L6 / Homologous L6	0.907	0.042	-2.085	0.295
Heterologous L6 / Homologous L6	1.086	0.067	1.344	0.760

Table S3. Coefficients of the Gamma generalized additive model for location, scale and shape (GAMLSS) fitted to phenoloxidase activity. The mean response (μ) was modeled as a function of developmental stage and treatment, whereas dispersion (σ) was modeled as a function of developmental stage. Estimates are reported on the log scale.

Parameter	Estimate	SE	t-value	p-value	Significance
**Mu (mean)**					
(Intercept)	0.322	0.036	8.93	<0.001	***
Development (L5L6)	-0.285	0.036	-7.93	<0.001	***
Development (L6)	0.396	0.059	6.73	<0.001	***
Development (L6L5)	-0.295	0.038	-7.78	<0.001	***
Treatment (Homologous)	-0.028	0.008	-3.37	<0.001	***
**Sigma (dispersion)**					
(Intercept)	-1.228	0.085	-14.42	<0.001	***
Development (L5L6)	-2.333	0.132	-17.65	<0.001	***
Development (L6)	0.194	0.125	1.56	0.121	
Development (L6L5)	-2.303	0.301	-7.65	<0.001	***

Table S4. Estimated marginal means for phenoloxidase activity obtained from the Gamma GAMLSS model. Means are presented with standard errors (SE) and 95% confidence intervals. Pairwise comparisons were performed using estimated marginal means with Tukey-adjusted P-values. Groups sharing the same letter are not significantly different (P > 0.05).

Treatment	Development	Estimated mean	SE	95% CI	Tukey group
Homologous	L6L5	0.959	0.129	0.665–1.38	ab
Homologous	L5L6	0.998	0.038	0.898–1.11	a
Heterologous	L6L5	1.027	0.13	0.726–1.45	ab
Heterologous	L5L6	1.069	0.052	0.936–1.22	a
Homologous	L5	1.314	0.046	1.195–1.44	b
Heterologous	L5	1.407	0.061	1.251–1.58	b
Homologous	L6	1.957	0.069	1.777–2.15	c
Heterologous	L6	2.096	0.097	1.847–2.38	c

Table S5. Model coefficent for lytic activity model.

Parameter	Estimate	SE	t-value	p-value	Significance
**Mu (mean)**					
(Intercept)	0.504	0.014	37.316	<0.00001	***
Development (L6)	0.154	0.019	7.937	<0.00001	***
Treatment (Heterologous)	-0.010	0.019	-0.545	0.586	
Treatment (Homologous)	-0.034	0.018	-1.928	0.056	*
**Sigma (dispersion)**					
(Intercept)	-2.542	0.073	-34.960	<0.001	***
Development (L6 vs L5)	0.741	0.108	6.864	<0.001	***

Table S6. Pairwise comparisons of lytic activity values between treatments.

Comparison	ratio	SE	z.ratio	p-value
Control L5 / Heterologous L5	0.957	0.026	-1.598	0.600
Control L5 / Homologous L5	1.006	0.019	0.301	1.000
**Control L5 / Control L6**	**0.867**	**0.015**	**-8.058**	**<.0001**
**Control L5 / Heterologous L6**	**0.830**	**0.027**	**-5.714**	**<.0001**
**Control L5 / Homologous L6**	**0.872**	**0.022**	**-5.416**	**<.0001**
Heterologous L5 / Homologous L5	1.050	0.026	1.958	0.367
**Heterologous L5 / Control L6**	**0.905**	**0.029**	**-3.070**	**0.026**
**Heterologous L5 / Heterologous L6**	**0.867**	**0.015**	**-8.058**	**<.0001**
**Heterologous L5 / Homologous L6**	**0.911**	**0.027**	**-3.114**	**0.023**
**Homologous L5 / Control L6**	**0.862**	**0.023**	**-5.574**	**<.0001**
**Homologous L5 / Heterologous L6**	**0.825**	**0.026**	**-6.105**	**<.0001**
**Homologous L5 / Homologous L6**	**0.867**	**0.015**	**-8.058**	**<.0001**
Control L6 / Heterologous L6	0.957	0.026	-1.598	0.600
Control L6 / Homologous L6	1.006	0.019	0.301	1.000
Heterologous L6 / Homologous L6	1.050	0.026	1.958	0.367

Table S7. Coefficients of the Gamma generalized additive model for location, scale and shape (GAMLSS) fitted to lytic activity. The mean response (μ) was modeled as a function of developmental stage and treatment, whereas dispersion (σ) was modeled as a function of developmental stage. Estimates are reported on the log scale.

Parameter	Estimate	SE	t-value	p-value	Significance
**Mu (mean)**					
(Intercept)	0.475	0.009	53.02	<0.001	***
Development (L5L6)	-0.476	0.009	-53.27	<0.001	***
Development (L6)	0.193	0.024	8.05	<0.001	***
Development (L6L5)	-0.482	0.019	-25.6	<0.001	***
Treatment (Homologous)	-0.002	0.004	-0.42	0.675	
**Sigma (dispersion)**					
(Intercept)	-2.64	0.086	-30.58	<0.001	***
Development (L5L6)	-1.666	0.132	-12.59	<0.001	***
Development (L6)	0.877	0.126	6.97	<0.001	***
Development (L6L5)	-0.564	0.301	-1.87	0.063	

Table S8. Estimated marginal means for lytic activity obtained from the Gamma GAMLSS model. Means are presented with standard errors (SE) and 95% confidence intervals. Pairwise comparisons were performed using estimated marginal means with Tukey-adjusted P-values. Groups sharing the same letter are not significantly different (P > 0.05).

Treatment	Development	Estimated mean	SE	95% CI	Tukey group
Homologous	L6L5	0.956	0.054	0.820–1.120	a
Homologous	L5L6	0.99	0.014	0.953–1.030	a
Heterologous	L6L5	0.992	0.053	0.857–1.150	a
Heterologous	L5L6	1.028	0.02	0.975–1.080	a
Homologous	L5	1.588	0.02	1.535–1.640	b
Heterologous	L5	1.648	0.029	1.571–1.730	b
Homologous	L6	1.895	0.024	1.831–1.960	c
Heterologous	L6	1.967	0.037	1.870–2.070	c

Biology Open • Supplementary information

Table S9. Model coefficent for total antioxidant activity model.

Parameter	Estimate	SE	t-value	p-value	Significance
**Mu (mean)**					
(Intercept – L5 Heterologous)	5.416	0.127	42.606	<0.0001	***
Development (L6 vs L5)	-1.738	0.123	-14.167	<0.0001	***
Treatment (Heterologous)	-0.115	0.050	-2.278	0.025	*
Treatment (Homologous)	0.030	0.050	0.604	0.547	
**Sigma (dispersion)**					
(Intercept)	-0.152	0.090	-1.679	0.096	.
Development (L6 vs L5)	-1.763	0.136	-12.992	<2e-16	***

Table S10. Pairwise comparisons of total antioxidant activity values between treatments.

Comparison	ratio	SE	z.ratio	p-value
**Control L5 / Heterologous L5**	**0.620**	**0.101**	**-2.942**	**0.038**
**Control L5 / Homologous L5**	**0.571**	**0.094**	**-3.409**	**0.009**
**Control L5 / Control L6**	**7.394**	**0.617**	**23.956**	**<.0001**
**Control L5 / Heterologous L6**	**4.585**	**0.909**	**7.676**	**<.0001**
**Control L5 / Homologous L6**	**4.225**	**0.839**	**7.253**	**<.0001**
Heterologous L5 / Homologous L5	0.921	0.072	-1.043	0.903
**Heterologous L5 / Control L6**	**11.924**	**1.970**	**14.979**	**<.0001**
**Heterologous L5 / Heterologous L6**	**7.394**	**0.617**	**23.956**	**<.0001**
**Heterologous L5 / Homologous L6**	**6.813**	**0.767**	**17.042**	**<.0001**
**Homologous L5 / Control L6**	**12.941**	**2.180**	**15.192**	**<.0001**
**Homologous L5 / Heterologous L6**	**8.025**	**0.935**	**17.865**	**<.0001**
**Homologous L5 / Homologous L6**	**7.394**	**0.617**	**23.956**	**<.0001**
**Control L6 / Heterologous L6**	**0.620**	**0.101**	**-2.942**	**0.038**
**Control L6 / Homologous L6**	**0.571**	**0.094**	**-3.409**	**0.009**
Heterologous L6 / Homologous L6	0.921	0.072	-1.043	0.903

Biology Open • Supplementary information

Table S11. Model coefficients for the total antioxidant capacity (TAC) model.

Parameter	Estimate	SE	t-value	p-value	Significance
**Mu (mean)**					
(Intercept – L5 Heterologous)	5.660	0.076	74.349	<0.0001	***
Development (L5L6 vs L5)	1.085	0.130	8.359	<0.0001	***
Development (L6 vs L5)	-2.095	0.077	-27.064	<0.0001	***
Development (L6L5 vs L5)	0.511	0.293	1.746	0.0838	.
Treatment (Homologous vs Heterologous)	0.117	0.052	2.254	0.0262	*
**Sigma (dispersion)**					
(Intercept)	-0.868	0.118	-7.349	<0.0001	***
Development (L5L6 vs L5)	0.486	0.157	3.089	0.0026	**
Development (L6 vs L5)	-0.932	0.170	-5.474	<0.0001	***
Development (L6L5 vs L5)	0.501	0.294	1.706	0.0911	.

Table S12. Estimated marginal means of total antioxidant capacity (TAC) for each treatment × developmental stage combination. Different letters indicate significant differences among groups according to Sidak-adjusted pairwise comparisons ($\alpha = 0.05$). Confidence intervals are backtransformed from the log scale.

Treatment	Development	Estimated mean	SE	95% CI	Group
Heterologous	L6	36.7	3.31	28.6–46.9	a
Homologous	L6	36.7	3.77	27.7–48.5	a
Heterologous	L5	303.1	26.9	237.9–386.1	b
Homologous	L5	303.4	31.6	228.5–403.0	b
Heterologous	L6L5	478.8	81.6	300.9–762.0	b
Homologous	L6L5	479.4	94.1	280.7–818.7	bc
Heterologous	L5L6	916.3	75.0	732.9–1145.6	cd
Homologous	L5L6	917.4	93.4	695.0–1211.0	d

Table S13. Model coefficent for catalase model.

Parameter	Estimate	SE	t-value	p-value	Significance
**Mu (mean)**					
(Intercept)	6.643	0.205	32.327	< 0.0001	***
Development (L6)	0.656	0.197	3.332	0.001	**
Treatment (Heterologous)	-0.391	0.266	-1.472	0.144	
Treatment (Homologous)	-0.021	0.231	-0.089	0.929	
**Sigma (dispersion)**					
(Intercept)	0.150	0.094	1.606	0.111	
Treatment (Heterologous)	-0.017	0.140	-0.125	0.901	
Treatment (Homologous)	-0.241	0.138	-1.745	0.084	.

Table S14. Pairwise comparisons of catalase values between treatments.

Comparison	ratio	SE	z.ratio	p-value
Control L5 / Heterologous L5	1.478	0.393	1.472	0.683
Control L5 / Homologous L5	1.021	0.236	0.089	1.000
**Control L5 / Control L6**	**0.519**	**0.102**	**-3.332**	**0.011**
Control L5 / Heterologous L6	0.767	0.255	-0.799	0.968
Control L5 / Homologous L6	0.530	0.159	-2.117	0.278
Heterologous L5 / Homologous L5	0.691	0.169	-1.514	0.655
**Heterologous L5 / Control L6**	**0.351**	**0.116**	**-3.179**	**0.019**
**Heterologous L5 / Heterologous L6**	**0.519**	**0.102**	**-3.332**	**0.011**
**Heterologous L5 / Homologous L6**	**0.358**	**0.111**	**-3.321**	**0.012**
Homologous L5 / Control L6	0.508	0.156	-2.200	0.238
Homologous L5 / Heterologous L6	0.751	0.240	-0.896	0.948
**Homologous L5 / Homologous L6**	**0.519**	**0.102**	**-3.332**	**0.011**
Control L6 / Heterologous L6	1.478	0.393	1.472	0.683
Control L6 / Homologous L6	1.021	0.236	0.089	1.000
Heterologous L6 / Homologous L6	0.691	0.169	-1.514	0.655

Table S15. Model coefficients for the catalase activity model.

Parameter	Estimate	SE	t-value	p-value	Significance
**Mu (mean)**					
(Intercept – L5 Heterologous)	6.224	0.204	30.547	<0.0001	***
Development (L5L6 vs L5)	1.237	0.217	5.695	<0.0001	***
Development (L6 vs L5)	0.709	0.241	2.942	0.0040	**
Development (L6L5 vs L5)	1.509	0.264	5.713	<0.0001	***
Treatment (Homologous vs Heterologous)	0.372	0.164	2.272	0.0250	*
**Sigma (dispersion)**					
(Intercept)	0.108	0.102	1.058	0.2923	
Development (L5L6 vs L5)	-0.471	0.148	-3.195	0.0018	**
Development (L6 vs L5)	-0.176	0.145	-1.209	0.2294	
Development (L6L5 vs L5)	-0.996	0.299	-3.333	0.0012	**

Table S16. Estimated marginal means of catalase activity for each treatment × developmental stage combination. Different letters indicate significant differences among groups according to Sidak-adjusted pairwise comparisons (α = 0.05). Confidence intervals are backtransformed from the log scale.

Treatment	Development	Estimated mean	SE	95% CI	Group
Heterologous	L5	503	92.2	305–829	a
Homologous	L5	726	118.0	467–1130	ab
Heterologous	L6	1034	192.0	624–1714	bc
Homologous	L6	1493	235.0	972–2292	cd
Heterologous	L5L6	1743	316.0	1063–2858	cd
Heterologous	L6L5	2281	937.0	744–6990	bcd
Homologous	L5L6	2516	397.0	1637–3867	d
Homologous	L6L5	3294	1470.0	975–11126	cd

Biology Open • Supplementary information

Table S17. Model coefficent for superoxide dismutase model.

Parameter	Estimate	SE	t-value	p-value	Significance
**Mu (mean)**					
(Intercept)	4.549	0.016	289.621	<0.0001	***
Development (L6 vs L5)	-0.334	0.050	-6.669	<0.0001	***
Treatment (Heterologous)	-0.007	0.022	-0.323	0.747	
Treatment (Homologous)	-0.015	0.022	-0.693	0.490	
**Sigma (dispersion)**					
(Intercept)	-2.677	0.094	-28.490	<0.0001	***
Development (L6 vs L5)	1.67716	0.13259	12.65	<0.0001	***

Table S18. Pairwise comparisons of superoxide dismutase values between treatments.

Comparison	ratio	SE	z.ratio	p-value
Control L5 / Heterologous L5	1.09	0.0693	1.43	0.7087
**Control L5 / Homologous L5**	**1.16**	**0.0572**	**2.982**	**0.034**
**Control L5 / Control L6**	**1.28**	**0.0504**	**6.371**	**<.0001**
**Control L5 / Heterologous L6**	**1.41**	**0.108**	**4.43**	**0.0001**
**Control L5 / Homologous L6**	**1.49**	**0.0974**	**6.073**	**<.0001**
Heterologous L5 / Homologous L5	1.06	0.0788	0.762	0.9738
Heterologous L5 / Control L6	1.17	0.0845	2.216	0.2301
**Heterologous L5 / Heterologous L6**	**1.28**	**0.0504**	**6.371**	**<.0001**
**Heterologous L5 / Homologous L6**	**1.36**	**0.114**	**3.662**	**0.0034**
Homologous L5 / Control L6	1.11	0.0672	1.697	0.5334
Homologous L5 / Heterologous L6	1.21	0.103	2.289	0.1983
**Homologous L5 / Homologous L6**	**1.28**	**0.0504**	**6.371**	**<.0001**
Control L6 / Heterologous L6	1.09	0.0693	1.43	0.7087
**Control L6 / Homologous L6**	**1.16**	**0.0572**	**2.982**	**0.034**
Heterologous L6 / Homologous L6	1.06	0.0788	0.762	0.9738

Biology Open • Supplementary information

Table S19. Model coefficients for the superoxide dismutase (SOD) activity model.

Parameter	Estimate	SE	t-value	p-value	Significance
**Mu (mean)**					
(Intercept – L5 Heterologous)	4.547	0.016	277.617	<0.0001	***
Development (L5L6 vs L5)	0.410	0.110	3.739	0.0003	***
Development (L6 vs L5)	-0.434	0.072	-6.008	<0.0001	***
Development (L6L5 vs L5)	0.721	0.059	12.205	<0.0001	***
Treatment (Homologous vs Heterologous)	-0.011	0.022	-0.505	0.6149	
**Sigma (dispersion)**					
(Intercept)	-2.661	0.115	-23.170	<0.0001	***
Development (L5L6 vs L5)	2.223	0.161	13.810	<0.0001	***
Development (L6 vs L5)	1.798	0.164	10.990	<0.0001	***
Development (L6L5 vs L5)	0.688	0.310	2.220	0.0285	*

Table S20. Estimated marginal means of superoxide dismutase (SOD) activity for each treatment × developmental stage combination. Different letters indicate significant differences among groups according to Sidak-adjusted pairwise comparisons (α = 0.05). Confidence intervals are backtransformed from the log scale.

Treatment	Development	Estimated mean	SE	95% CI	Group
Homologous	L6	55.9	4.83	44.1–70.7	a
Heterologous	L6	65.7	4.72	54.0–79.9	ab
Homologous	L5	83.8	7.09	66.5–105.5	bc
Heterologous	L5	98.5	6.71	81.8–118.6	cd
Homologous	L5L6	135.7	12.7	105.2–175.0	de
Heterologous	L5L6	159.5	10.4	133.4–190.6	e
Homologous	L6L5	165.0	25.0	109.1–249.6	de
Heterologous	L6L5	194.0	24.6	137.2–274.2	e

Biology Open • Supplementary information

Table S21. Model coefficent for hydrogen peroxide model.

Parameter	Estimate	SE	t-value	p-value	Significance
**Mu (mean)**					
(Intercept – L5 Heterologous)	6.743	0.214	31.480	<0.0001	***
Development (L6 vs L5)	1.485	0.206	7.207	<0.0001	***
Treatment (Heterologous)	-0.632	0.352	-1.792	0.076	.
Treatment (Homologous)	-0.502	0.238	-2.108	0.038	*
**Sigma (dispersion)**					
(Intercept)	0.194	0.103	1.883	0.063	.
Treatment (Heterologous)	0.187	0.153	1.226	0.223	
Treatment (Homologous)	-0.532	0.155	-3.430	0.001	***

Table S22. Pairwise comparisons of hydrogen peroxide values between treatments.

Comparison	ratio	SE	z.ratio	p-value
Control L5 / Heterologous L5	1.882	0.663	1.794	0.469
Control L5 / Homologous L5	1.655	0.395	2.115	0.280
**Control L5 / Control L6**	**0.226**	**0.047**	**-7.215**	**<.0001**
Control L5 / Heterologous L6	0.426	0.168	-2.167	0.253
**Control L5 / Homologous L6**	**0.375**	**0.104**	**-3.528**	**0.006**
Heterologous L5 / Homologous L5	0.880	0.273	-0.414	0.998
**Heterologous L5 / Control L6**	**0.120**	**0.051**	**-5.016**	**<.0001**
**Heterologous L5 / Heterologous L6**	**0.226**	**0.047**	**-7.215**	**<.0001**
**Heterologous L5 / Homologous L6**	**0.199**	**0.071**	**-4.504**	**0.000**
**Homologous L5 / Control L6**	**0.137**	**0.048**	**-5.720**	**<.0001**
**Homologous L5 / Heterologous L6**	**0.257**	**0.099**	**-3.520**	**0.006**
**Homologous L5 / Homologous L6**	**0.226**	**0.047**	**-7.215**	**<.0001**
Control L6 / Heterologous L6	1.882	0.663	1.794	0.469
Control L6 / Homologous L6	1.655	0.395	2.115	0.280
Heterologous L6 / Homologous L6	0.880	0.273	-0.414	0.998

Table S23. Model coefficients for the hydrogen peroxide (H_2O_2) model.

Parameter	Estimate	SE	t-value	p-value	Significance
**Mu (mean)**					
(Intercept – L5 Heterologous)	5.906	0.257	22.944	<0.0001	***
Development (L5L6 vs L5)	-2.557	0.288	-8.877	<0.0001	***
Development (L6 vs L5)	1.565	0.279	5.611	<0.0001	***
Development (L6L5 vs L5)	-3.161	0.585	-5.407	<0.0001	***
Treatment (Homologous vs Heterologous)	0.416	0.195	2.138	0.0349	*
**Sigma (dispersion)**					
(Intercept)	0.294	0.106	2.765	0.0068	**
Development (L5L6 vs L5)	-0.306	0.146	-2.090	0.0392	*
Development (L6 vs L5)	-0.508	0.153	-3.311	0.0013	**
Development (L6L5 vs L5)	-0.043	0.265	-0.162	0.8718	

Biology Open • Supplementary information

Table S24. Estimated marginal means of hydrogen peroxide (H_2O_2) concentration for each treatment × developmental stage combination. Different letters indicate significant differences among groups according to Sidak-adjusted pairwise comparisons (α = 0.05). Confidence intervals are backtransformed from the log scale.

Treatment	Development	Estimated mean	SE	95% CI	Group
Heterologous	L6L5	15.6	8.36	3.60–67.3	a
Homologous	L6L5	21.5	12.5	4.41–104.6	a
Heterologous	L5L6	32.7	7.61	17.31–61.7	a
Homologous	L5L6	45.1	7.12	29.30–69.3	a
Heterologous	L5	343.3	78.7	183.66–641.6	b
Homologous	L5	473.6	83.0	293.66–763.7	b
Heterologous	L6	1889.1	450.0	986.36–3618.1	c
Homologous	L6	2606.0	424.0	1671.77–4062.5	c

Dataset 1.

Available for download at
https://journals.biologists.com/bio/article-lookup/doi/10.1242/bio.062421#supplementary-data

Dataset 2.

Available for download at
https://journals.biologists.com/bio/article-lookup/doi/10.1242/bio.062421#supplementary-data

Dataset 3.

Available for download at
https://journals.biologists.com/bio/article-lookup/doi/10.1242/bio.062421#supplementary-data
