# Supplementary material for: Is the developmental stage important for the plasticity of transgenerational immune priming?
Source: Biol Open. 2026 Aug 27;15(8):bio062421. doi: 10.1242/bio.062421 (PMC13580588; doi:10.1242/bio.062421)
Supplement: Supplementary file 2 [file biolopen-15-062421_review_history.pdf]

IS THE DEVELOPMENTAL STAGE IMPORTANT FOR THE PLASTICITY OF TRANSGENERATIONAL IMMUNE PRIMING?

Fernanda Vázquez-Fuerte, Angélica González-Hernández, Juan Carlos Torres Guzmán and Jorge Contreras-Garduño
10.1242/bio.062421

Editor: Kendra J. Greenlee

Review timeline
Original submission: 12 December 2025
Editorial decision: 26 December 2025
First revision received: 22 March 2026
Editorial decision: 2 April 2026
Second revision received: 10 July 2026
Accepted: 16 July 2026

Original submission

First decision letter

MS ID#: bio.062421

MS Title: Is the Developmental Stage Important for the Plasticity of Transgenerational Immune Priming?

Authors: Fernanda Vázquez-Fuerte; Angélica González-Hernández; Juan Carlos Torres Guzmán; Jorge Contreras-Garduño

I have now reached a decision on the above manuscript.

The reviewer reports are shown at the bottom of this email.

As you will see, the reviewers raised a number of substantial criticisms that prevent me from accepting the paper at this stage.

They suggest, however, that a revised version might prove acceptable, if you can address their concerns. If you think that you can deal satisfactorily with the criticisms on revision, I would be pleased to see a revised manuscript. In particular, the concerns raised by Reviewer 1 in the section "Experimental Quality" and by Reviewer 2 about the missing data/figures should be addressed along with the minor comments.

At this stage, we also ask you to ensure your manuscript complies with our formatting guidelines. Provided you are able to fully address the referees' comments, we are positive about publication of your paper (we accept over 95% of revision submissions) and therefore hope you won't mind any extra work involved in reformatting your manuscript at this point.

Please upload both a 'clean' version of your Word file, along with a highlighted version clearly showing where you have made changes in the revised manuscript. Please avoid using 'Track changes' in Word files as these are lost in PDF conversion.

© 2026. Published by The Company of Biologists under the terms of the Creative Commons Attribution License (https://creativecommons.org/licenses/by/4.0/).

I should be grateful if you would also provide a point-by-point response detailing how you have dealt with the points raised by the reviewers in the 'Response to Reviewers' box. Please attend to all of the reviewers' comments. If you do not agree with any of their criticisms or suggestions please explain clearly why this is so.

Reviewer 1

Comments for the author

In this paper by Vázquez-Fuerte et al., the possibility of immune memory passed down from one generation to another is discussed using Spodoptera frugiperda and infecting with the fungus, Metarhizium brunneum, in both the F0 generation and their offspring. They found that, if the parental generation was infected at the L5 stage, their offspring would have better survival if also challenged at the L5 stage compared to those whose parents were not infected, showing immune memory passed down from the parental generation. They also saw significant differences between immune markers at various stages, but not between treatments. Overall, this is fascinating work in physiology of insects that could have impacts in control methods in the future. However, some rearranging and clarifications need to be made in the manuscript before publication. Please see the Line-by-line section for specific comments and questions.

Experimental quality: The experiments in this paper are robust and sound with some notable exceptions: none of the F1 generation were controls. This would have been valuable information when comparing survival and larval development. Instead, there is no negative control in the second half of the experiment. Additionally, there seems to be no analysis of the immune markers in the mixed-stage treatment groups (L5→L6; L6→L5; etc.), which seems like it would be important information in determining how the immune system is responding in the different treatment groups. Statistical analyses seem appropriate, but the significant results from the tests should be represented in the graphs as well as the text.

Reproducibility: The experiments performed in this paper, for the most part, seem reproducible. Sample sizes seem adequate (some were likely limited to how many offspring were produced by the F0 generation). Clarification on certain aspects, listed in Line-by-line, will help with reproducibility.

Completeness: This manuscript is technically complete as is, and the conclusions are supported by the data. This reviewer would love to see the analysis of the immune markers in the mixed-stage treatment groups as well as a negative control for the second generation, but neither of those would disprove what is in the manuscript. They would only add deeper understanding.

Scholarship: The authors discuss relevant literature in their field in order to put their work into context, both supporting and opposing.

Line-by-line:

Abstract:

Line 20-21: There is not a clear connection between the first two sentences. Please revise.

Line 24-25: Include common names or type of organism here if possible.

Introduction:

Line 52-54: This sentence is a lot of jargon all at once. Please consider revising.

Line 60-61: This sentence is missing a few commas-shoudl be "[...] immune priming, and, if […] effective, can cause.."

Line 94: There should not be a comma after "both"

© 2026. Published by The Company of Biologists under the terms of the Creative Commons Attribution License (https://creativecommons.org/licenses/by/4.0/).

General: There are a lot of literature comparisons in the intro, which feel like a better fit for discussion. The authors might not need all the explanations of each paper cited, rather mention relevant information in the intro then explain the study in order to compare to your own in the discussion.

Methods:

Line 166-175: The model organism should be introduced in the introduction with specifics about collection and/or rearing in the methods. Please move 166-175 to the introduction (can be added around line 130).

Line 177-179: Why the change in photoperiod in the different life stages?

Line 181: Sub-sub headings for this section would be beneficial!

Line 183-185: Belongs in the next paragraph. Talk about the strains of pathogens used, then discuss the doses.

Line 183: Remove "pilot," as this is not necessary here.

Line 184: Is this sublethal if it is lethal to a quarter of the population? This reviewer suggests changing the terminology to "LD100" for the "lethal" dose and "LD25" for the sublethal for clarity.

Line 197: This "Hamilton" citation is incomplete, as it should have the CAS#, the company, and the location of the company (unless this is a paper, in which case it needs a year).

Line 213: When the authors say "randomly paired"-still in the same group? Or were control mixed with treatment to determine the effect of paternal vs maternal immune response? Please clarify.

Line 224: Please define the abbreviations L5R vs L5C vs L5C-L6R. I assume C is control and R is treatment, but it is a bit unclear (what does the R stand for? It looks like you use "F" instead in Figure 3). Please also very clearly define homologous vs heterologous, since this could refer to the mixed treatment or the mixed larval stages. Please then briefly remind the reader in the results section, since these terms are used often.

Figure 1: What are the "35" and "30" on the x axis? (This figure is very helpful, by the way!!)

Line 298: The spacing is odd for these headings-please adjust formatting for final submission.

Line 298 & 311: The phenoloxidase and Lytic activity methods are essentially the same. Could these be combined? Or the lytic activity could say "followed the same protocol of phenoloxidase" after the preparation steps.

Line 350-351: Why was the 1X working solution added twice to the first well? A table might be beneficial here to represent the standard curves (and maybe some of the other solutions as well?).

Results

Figure 3: How is survival probability calculated compared to survival? Also, if possible, represent significance on the graph and provide sample sizes in the caption.

Line 480: Please avoid contractions.

Figure 4: This reviewer does not understand the point of this figure, since it shows the exact same data as figure 3 minus 2 lines. Refer the reader to figure 3 and remove this one.

General thought: Were there any F1 offspring that were just control? This would be important when comparing survival.

© 2026. Published by The Company of Biologists under the terms of the Creative Commons Attribution License (https://creativecommons.org/licenses/by/4.0/).

Figure 5: Formatting seems to have shifted here. Is the legend any different from the x-axis labels? If not, the legend and the different colors (while pretty!) are not necessary. The figure caption needs to be corrected as well-5b should be from L6, not L5 and L6. Could you also include the sample size in the figure caption? It looks like only 4/group.

Line 595: This contains a comma splice. Consider changing to "... treatment in L6, which were not significantly different."

Figure 6 & 7: While putting "a) phenoloxidase" and "b) lytic activity" as the titles of this graph is helpful (in my opinion), it is general practice to just have that information in the caption. This reviewer personally finds that informative, but it is usually not done. Please add stats to these as well (i.e. putting letters or stars on the graphs for significantly different groups).

Line 623-625: Nothing significant between treatments-are none of these good indicators of immune memory? Shouldn't L6 larvae that came from L5 challenged parents (L5→L6) have the immune memory? Are these not included in this part of the study? And shouldn't the levels of the immune markers be different in L5 for the different treatments? Homologous should have immunity, and heterologous should have the same response as L6 due to lack of immune memory, no? Or do they lose the immune memory at L6? This needs to be discussed (in the discussion and in the results) more explicitly.

Discussion

Line 648-662: The majority of this paragraph belongs in methods and can be inserted when talking about using the two different fluorescent probes (around line 189). While it is great that you did this method of checking for infection clearance to determine immune memory versus vertical transmission, I don't see this as a main point that should be discussed in the discussion. The discussion is to dive deeper into what your results meant and how they compared to other studies, not to explain reasoning for methodology, even if it is rare. That should be in methods or discussion. The only reason this should be in the discussion is to explain your results or why your results were different from other groups with similar experiments (i.e the other group did not check for infection clearance, which may have affected their results to make them different from yours: 695-897 would be a perfect place to put line 653-662). Please consider reorganizing/moving this section to introduction and/or methods.

Line 668: Please make it clear this is another paper you are talking about. Also, please compare your work in more detail with each reference you are summarizing-this isn't a review paper, so be sure to insert how each one relates to your results.

Line 698: Spodoptera should be italicized

Line 698-700: This would be helpful information in the introduction and/or methods as to why you are focusing on L5 & L6.

<u>Reviewer 2</u>

Comments for the author

Vazquez-Fuente and colleagues propose a study of immune response memory using Spodoptera frugiperda infected with the fungus Metarhizium brunneum as a model system. The study addresses the role of developmental plasticity in the mounting of immune memory in arthropods. Using a rigorous methodology, the authors showed that immune priming works at the L5 stage. Secondly, using a multitude of metabolic processes, the authors show that a trade-off between immune protection and energy for development impacts the efficacy of immune priming.

Overall, this is a clear and comprehensive manuscript exploring a very important and novel question of immune priming and memory in arthropods. I have suggested below some modifications to improve certain parts and figures of the manuscript.

© 2026. Published by The Company of Biologists under the terms of the Creative Commons Attribution License (https://creativecommons.org/licenses/by/4.0/).

Major comments:

Some parts of the results text related to development have missing data and figures.

Line 520-525: The authors must provide the data and figures related to developmental time at larval, pupal, and adult stages for the different immunisation plans performed here. The authors provide statistics on data in the text without accompanying data or figures.

Minor comments:

Line 116: What is the classification of "nucleopolyhedrovirus"?

Line 122: Could you provide the full name of SpexNPV and its classification?

General comments on the introduction: The concept of immune priming was well established and comprehensive. The authors introduce the methods, etc. However, we need some introduction to the pathogen used in the study. Why did the authors choose Metarhizium brunneum? What type of immune response is induced by the infection? Is the S. frugiperda-M. brunneum system a good model to study the immune system (natural infection?)?

Line 202: No Figure S2 is attached to the manuscript.

Lines 213-214: For the random pairing, were control and memory groups mixed, or did the authors mix males and females? This sentence could be clearer for the reader.

Line 298: Phenoloxidase—is this an assay, a measurement, or something else? Please clarify.

Line 229: Same comment—could you clarify?

Figure 2: The figure legend is scarce and incomplete for the reader to understand the figure. The authors need to provide a scale and a legend for the pictures (in both legend and figure). The authors need to provide a positive control for GFP—ideally a picture with GFP-positive infection, and then after clearance. That would be helpful.

Figure 3: Statistical analyses, while clearly stated in the results text, are missing in both the figure and legend. Please add these.

Lines 481-482: Reference the figure supporting this statement.

Figure 4: Like Figure 3, statistics are needed for Figure 4 in both the figure and the legend.

Lines 503-509: Authors need to reference the figure associated with the statistics.

Figure 5: The authors added the Kruskal-Wallis test statistics in the figure, but no details are given in the legend, so it is unclear what the test relates to. The legend needs to be more complete and descriptive, with colour references and statistics included.

Line 552: Could the authors mention the values for phenoloxidase activity?

Figure 6: Statistics are needed in the figure and legend. Make the whiskers more visible. Describe the colours in the legend. Why did the authors not keep the colours used originally for each immunisation-challenge plan?

Figure 7: Similar comments to Figure 6—see above.

Discussion: I really appreciated the discussion. The placement in the literature is good, but I would appreciate it if the authors could refer more to their own conclusions and observations. To help with that, I suggest that the authors reference figures in the discussion. For example, at line 662, the authors should reference their figure. The same applies to line 686 and each paragraph thereafter.

© 2026. Published by The Company of Biologists under the terms of the Creative Commons Attribution License (https://creativecommons.org/licenses/by/4.0/).

Reviewer's Responses to Questions

Experimental quality

Does each figure have the proper controls?
If 'No', please indicate reasons in Comments for Author box below.
Reviewer #1:
- Yes

Reviewer #2:
- Yes

Were the data analyzed using appropriate statistical tests?
If 'No', please indicate reasons in Comments for Author box below.
Reviewer #1:
- Yes

Reviewer #2:
- Yes

Reproducibility

Were experiments performed using adequate number of biological replicates?
If 'No', please indicate reasons in Comments for Author box below.
Reviewer #1:
- Yes

Reviewer #2:
- Yes

Does the methods section provide sufficient detail to permit reproducibility?
If 'No', please indicate reasons in Comments for Author box below.
Reviewer #1:
- Yes

Reviewer #2:
- Yes

Completeness

Are the manuscript's conclusions supported by the data?
If 'No', please indicate reasons in Comments for Author box below.
Reviewer #1:
- Yes

Reviewer #2:
- No

Scholarship

Do the authors cite and discuss the merits of data that would argue for and against their conclusion?

© 2026. Published by The Company of Biologists under the terms of the Creative Commons Attribution License (https://creativecommons.org/licenses/by/4.0/).

If 'No', please indicate reasons in Comments for Author box below.
Reviewer #1:
- Yes

Reviewer #2:
- Yes

Does the manuscript title & abstract accurately reflect the contents of the manuscript, without hyperbole?
If 'No', please indicate reasons in Comments for Author box below.
Reviewer #1:
- Yes

Reviewer #2:
- Yes

First revision

Author response to reviewers' comments

We would like to thank you and the reviewers for your constructive feedback on our manuscript number: **ID#: bio.062421** now entitled: **"I don't want to grow up: the developmental stage may impair the transgenerational immune priming"**. We have carefully considered each of your comments and made the necessary revisions accordingly. Below, we highlight in black the reviewers' comments and our response in blue. We are open to carrying out more changes if needed.

Reviewer 1: Please see attachment for proper formatting.

Summary: In this paper by Vázquez-Fuerte et al., the possibility of immune memory passed down from one generation to another is discussed using Spodoptera frugiperda and infecting with the fungus, Metarhizium brunneum, in both the F0 generation and their offspring. They found that, if the parental generation was infected at the L5 stage, their offspring would have better survival if also challenged at the L5 stage compared to those whose parents were not infected, showing immune memory passed down from the parental generation. They also saw significant differences between immune markers at various stages, but not between treatments. Overall, this is fascinating work in physiology of insects that could have impacts in control methods in the future. However, some rearranging and clarifications need to be made in the manuscript before publication. Please see the Line-by-line section for specific comments and questions.

Experimental quality: The experiments in this paper are robust and sound, with some notable exceptions: none of the F1 generation were controls. This would have been valuable information when comparing survival and larval development. Instead, there is no negative control in the second half of the experiment. Additionally, there seems to be no analysis of the immune markers in the mixed-stage treatment groups (L5→L6; L6→L5; etc.), which seems like it would be important information in determining how the immune system is responding in the different treatment groups. Statistical analyses seem appropriate, but the significant results from the tests should be represented in the graphs as well as the text.

Reproducibility: The experiments performed in this paper, for the most part, seem reproducible. Sample sizes seem adequate (some were likely limited to how many offspring were produced by the F0 generation). Clarification on certain aspects, listed in Line-by-line, will help with reproducibility.

Completeness: This manuscript is technically complete as is, and the conclusions are supported by the data. This reviewer would love to see the analysis of the immune markers in the mixed-stage

© 2026. Published by The Company of Biologists under the terms of the Creative Commons Attribution License (https://creativecommons.org/licenses/by/4.0/).

treatment groups as well as a negative control for the second generation, but neither of those would disprove what is in the manuscript. They would only add deeper understanding.

Scholarship: The authors discuss relevant literature in their field in order to put their work into context, both supporting and opposing.

Response: Thank you very much for all your comments. We respond line-by-line below in blue, and we marked our responses in yellow in the manuscript. We included the control group in F1 as you suggested and this comparsision provide more support to our results (see for example lines 758-770, 781-784). Unfortunately, we have not finished the analyze for L5→L6; L6→L5 but if you think is determinant, we can do our best to finish this experiment soon. We are open to carry out more experiments and make more changes to our MS if it is needed.

Abstract:

Line 20-21: There is not a clear connection between the first two sentences. Please revise.

Response: We delete the first sentence (lines 20-21).

Line 24-25: Include common names or type of organism here if possible.

Response: Included in lines 23 and 24.

Introduction:

Line 52-54: This sentence is a lot of jargon all at once. Please consider revising.

Response: In lines 48-50 we have rephrased this sentence to reduce jargon while preserving the original meaning.

Line 60-61: This sentence is missing a few commas-shoudl be "[...] immune priming, and, if [...] effective, can cause.."

Response: We have added the suggested commas and revised the sentence to improve clarity (lines 55-58).

Line 94: There should not be a comma after "both"

Response: We have removed the comma after “both” (line 86).

General: There are a lot of literature comparisons in the intro, which feel like a better fit for discussion. The authors might not need all the explanations of each paper cited, rather mention relevant information in the intro then explain the study in order to compare to your own in the discussion.

Response: We have revised the introduction to reduce extensive literature comparisons and detailed descriptions of previous studies. The new version focuses on the conceptual framework and knowledge gaps motivating our study, while detailed comparisons with previous work have been moved to the discussion.

Methods:

Line 166-175: The model organism should be introduced in the introduction with specifics about collection and/or rearing in the methods. Please move 166-175 to the introduction (can be added around line 130).

Response: We have moved the general biological description of *Spodoptera frugiperda* to the Introduction and retained only experimental rearing conditions in the Methods section. See lines 103-130.

© 2026. Published by The Company of Biologists under the terms of the Creative Commons Attribution License (https://creativecommons.org/licenses/by/4.0/).

Line 177-179: Why the change in photoperiod in the different life stages?

Response: The difference in photoperiod reflects the natural diurnal activity of larvae and the nocturnal behavior of adults. This has been clarified in the Methods section (lines 159-165).

Line 181: Sub-sub headings for this section would be beneficial!

Response: We have now added sub-subheadings throughout the Methods section to improve clarity.

Line 183-185: Belongs in the next paragraph. Talk about the strains of pathogens used, then discuss the doses.

Response: We have reorganized this section (lines 171-181).

Line 183: Remove "pilot," as this is not necessary here.

Response: We have removed the term as suggested.

Line 184: Is this sublethal if it is lethal to a quarter of the population? This reviewer suggests changing the terminology to "LD100" for the "lethal" dose and "LD25" for the sublethal for clarity.

Response: We agree that referring to a dose causing approximately 25% mortality as "sublethal" may be ambiguous. To improve clarity and precision, we have replaced the terms "lethal" and "sublethal" with LD100 and LD25, respectively, throughout the manuscript. For example, see lines 25, 26, 189-191 or 197.

Line 197: This "Hamilton" citation is incomplete, as it should have the CAS#, the company, and the location of the company (unless this is a paper, in which case it needs a year).

Response: The syringe information has been completed. Lines 186-187.

Line 213: When the authors say "randomly paired"-still in the same group? Or were control mixed with treatment to determine the effect of paternal vs maternal immune response? Please clarify.

Response: Individuals were randomly paired within the same treatment group (memory × memory or control × control). No crosses between treatments were performed, and paternal and maternal effects were not analyzed separately. The text has been clarified accordingly. Lines 202-206.

Line 224: Please define the abbreviations L5R vs L5C vs L5C-L6R. I assume C is control and R is treatment, but it is a bit unclear (what does the R stand for? It looks like you use "F" instead in Figure 3). Please also very clearly define homologous vs heterologous, since this could refer to the mixed treatment or the mixed larval stages. Please then briefly remind the reader in the results section, since these terms are used often.

Response: We have revised the nomenclature throughout the manuscript to ensure consistency and avoid ambiguity.
First, we now consistently use C to denote parental control injection (Tween) and F to denote fungal challenge (*Metarhizium brunneum*), replacing the previously inconsistent use of "R". Treatment codes therefore indicate both the parental treatment and the offspring treatment, as well as the larval stage at which each challenge was applied (e.g., L5F–L6F). Second, we clarify that the terms homologous and heterologous refer exclusively to the type of immune challenge, not to the larval stage. Homologous treatments indicate that both parents and offspring were challenged with the fungus (F–F), whereas heterologous treatments indicate that parents received a control injection and offspring were challenged with the fungus (C–F). The larval stage at challenge (L5 or L6) is treated as a separate experimental factor and is explicitly described as "same stage" or "different stage" throughout the Methods section.

Figure 1: What are the "35" and "30" on the x axis? (This figure is very helpful, by the way!!)

© 2026. Published by The Company of Biologists under the terms of the Creative Commons Attribution License (https://creativecommons.org/licenses/by/4.0/).

Response: These numbers indicate the duration (in days) of the life cycle recorded during the experiment. The parental (F0) generation lasted 35 days, whereas the offspring (F1) generation lasted 30 days. The x-axis was scaled accordingly to reflect the duration of each generation. We have included this information in the figure legend. This figure is now the figure 2. See lines 233-254, 260-268.

Line 298: The spacing is odd for these headings-please adjust formatting for final submission.

Response: Line 298: We have adjusted the spacing between sections.

Line 298 & 311: The phenoloxidase and Lytic activity methods are essentially the same. Could these be combined? Or the lytic activity could say "followed the same protocol of phenoloxidase" after the preparation steps.

Response: Although the phenoloxidase and lytic activity assays measure different immune parameters and require different reagent preparations, the incubation and plate-reading procedures are the same. To avoid redundancy, we revised the text so that the lytic activity assay now refers to the same incubation and reading protocol described for the phenoloxidase assay. See lines 309-311.

Line 350-351: Why was the 1X working solution added twice to the first well? A table might be beneficial here to represent the standard curves (and maybe some of the other solutions as well?).

Response: The first well corresponds to the blank of the standard curve and therefore contained only 25 µL of the 1X working solution (0 catalase). The previous wording did not clearly indicate that this well was used as the blank, which may have confused. We have revised the text to explicitly state this. Lines 340-347.

For clarity, the preparation of the catalase standards used to generate the standard curve is summarized below:

Catalase activity	Catalase stock	Catalase volume (µL)	1X working solution (µL)	Final volume (µL)
0 U	-	-	25	25
0.00625 U	1 U/mL	6.25	18.75	25
0.0125 U	1 U/mL	12.5	12.5	25
0.025 U	10 U/mL	2.5	22.5	25
0.05 U	10 U/mL	5	20	25
0.1 U	10 U/mL	10	15	25

Results

Figure 3: How is survival probability calculated compared to survival? Also, if possible, represent significance on the graph and provide sample sizes in the caption.

Response: In the figure, the y-axis represents the estimated survival probability calculated using the Kaplan-Meier estimator. Unlike simple survival proportions (i.e., the number of individuals remaining alive at a given time point), the Kaplan-Meier method estimates the cumulative probability of survival over time based on the number of individuals at risk and the mortality events observed. We have clarified this in the revised figure caption. See lines 465-486.

Line 480: Please avoid contractions.

Response: The contraction has been removed, and the sentence has been revised accordingly.

Figure 4: This reviewer does not understand the point of this figure, since it shows the exact same data as figure 3 minus 2 lines. Refer the reader to figure 3 and remove this one.

© 2026. Published by The Company of Biologists under the terms of the Creative Commons Attribution License (https://creativecommons.org/licenses/by/4.0/).

Response: We agree that the original Figure 4 was redundant with Figure 3. Following the reviewer's recommendation, the original figure has been removed. To improve clarity and better highlight the differences between developmental stages, we reorganized the survival analyses so that Figure 3 now presents the Kaplan-Meier survival curves for offspring challenged at the L5 stage, whereas Figure 4 presents the curves for offspring challenged at the L6 stage. This separation allows a clearer visualization of the patterns associated with each developmental stage. Lines 438-512.

General thought: Were there any F1 offspring that were just control? This would be important when comparing survival.

Response: We apologize for the lack of clarity in the previous version. F1 control groups were indeed included in the experiment, but were not clearly indicated in the manuscript. In the revised version, we have explicitly included the F1 control treatments (L5C–L5C and L6C–L6C), in which both parents and offspring were injected with Tween at the same developmental stage. These control groups are now reflected in the experimental design and described throughout the Results section.

Figure 5: Formatting seems to have shifted here. Is the legend any different from the x-axis labels? If not, the legend and the different colors (while pretty!) are not necessary. The figure caption needs to be corrected as well-5b should be from L6, not L5 and L6. Could you also include the sample size in the figure caption? It looks like only 4/group.

Response: The legend has been removed since the information was redundant with the x-axis labels, and the figure formatting has been corrected. We also revised the figure caption to clarify that panel b) corresponds to offspring from parents challenged at the L6 stage. Additionally, the sample sizes for each treatment group have now been included in the caption.

Line 595: This contains a comma splice. Consider changing to "... treatment in L6, which were not significantly different."

Response: This section has been restructured to incorporate the control treatment and improve the clarity of the results. As part of this revision, the sentence containing the comma splice has been corrected.

Figure 6 & 7: While putting "a) phenoloxidase" and "b) lytic activity" as the titles of this graph is helpful (in my opinion), it is general practice to just have that information in the caption. This reviewer personally finds that informative, but it is usually not done. Please add stats to these as well (i.e. putting letters or stars on the graphs for significantly different groups).

Response: The panel labels previously included in the graphs have been removed, and this information is now presented only in the figure captions. In addition, statistical differences among groups are now indicated in the graphs using letters corresponding to the results of the post hoc pairwise comparisons. Lines 516-799.

Line 623-625: Nothing significant between treatments-are none of these good indicators of immune memory? Shouldn't L6 larvae that came from L5 challenged parents (L5→L6) have the immune memory? Are these not included in this part of the study? And shouldn't the levels of the immune markers be different in L5 for the different treatments? Homologous should have immunity, and heterologous should have the same response as L6 due to lack of immune memory, no? Or do they lose the immune memory at L6? This needs to be discussed (in the discussion and in the results) more explicitly.

Response: In the antioxidant parameters analyzed (TAC, catalase, SOD, and H_2O_2), our results indicate that developmental stage explained most of the variation in these markers, whereas treatment effects were generally weak or absent. Although some parameters (e.g., TAC) showed differences between control and challenged groups, most comparisons between homologous and heterologous treatments were not significant within a developmental stage.This suggests that these oxidative stress markers primarily reflect physiological changes associated with development rather than treatment-specific immune priming. We agree that if immune memory were mediated through

© 2026. Published by The Company of Biologists under the terms of the Creative Commons Attribution License (https://creativecommons.org/licenses/by/4.0/).

these pathways, stronger differences between homologous and heterologous treatments might be expected.

Discussion

Line 648-662: The majority of this paragraph belongs in methods and can be inserted when talking about using the two different fluorescent probes (around line 189). While it is great that you did this method of checking for infection clearance to determine immune memory *versus* vertical transmission, I don't see this as a main point that should be discussed in the discussion. The discussion is to dive deeper into what your results meant and how they compared to other studies, not to explain reasoning for methodology, even if it is rare. That should be in methods or discussion. The only reason this should be in the discussion is to explain your results or why your results were different from other groups with similar experiments (i.e the other group did not check for infection clearance, which may have affected their results to make them different from yours: 695-897 would be a perfect place to put line 653-662). Please consider reorganizing/moving this section to introduction and/or methods.

Response: We agree that the explanation regarding infection clearance is primarily methodological. Therefore, in the revised manuscript, we have moved this section to the Methods, where the use of the fluorescent probes is described. Lines 212-231.

Line 668: Please make it clear this is another paper you are talking about. Also, please compare your work in more detail with each reference you are summarizing-this isn't a review paper, so be sure to insert how each one relates to your results.

Response: We have revised this section to clarify that we are referring to independent studies and to explicitly relate each reference to our findings.

Line 698: Spodoptera should be italicized

Response: The genus name *Spodoptera* has been italicized in the revised manuscript. Line 777.

Line 698-700: This would be helpful information in the introduction and/or methods as to why you are focusing on L5 & L6.

Response: We have clarified this point in the Introduction. Line 116-122.

Reviewer 2: Vazquez-Fuente and colleagues propose a study of immune response memory using Spodoptera frugiperda infected with the fungus Metarhizium brunneum as a model system. The study addresses the role of developmental plasticity in the mounting of immune memory in arthropods. Using a rigorous methodology, the authors showed that immune priming works at the L5 stage. Secondly, using a multitude of metabolic processes, the authors show that a trade-off between immune protection and energy for development impacts the efficacy of immune priming. Overall, this is a clear and comprehensive manuscript exploring a very important and novel question of immune priming and memory in arthropods. I have suggested below some modifications to improve certain parts and figures of the manuscript.

Major comments:

Some parts of the results text related to development have missing data and figures.

Line 520-525: The authors must provide the data and figures related to developmental time at larval, pupal, and adult stages for the different immunisation plans performed here. The authors provide statistics on data in the text without accompanying data or figures.

Response: We have revised this section to clarify the presentation of the developmental data. Larval developmental time, which showed significant differences among treatments, is reported in the text and discussed accordingly. For the pupal stage, no significant differences in developmental duration were detected among treatments; therefore, these results are reported only in the text

© 2026. Published by The Company of Biologists under the terms of the Creative Commons Attribution License (https://creativecommons.org/licenses/by/4.0/).

and the reference to a figure has been removed. Finally, adult-stage development was not included in the analysis because the focus of the experiment was on larval and pupal stages, where immune priming and developmental trade-offs are expected to occur. For adults, we were focused on obtain eggs, as this is a little bit difficult to obtain pair by pair. The manuscript has been revised to avoid referring to figures or datasets that are not included. We are open to carry out more experiments and make more changes to our MS if it is needed. In the MS, you will see our responses to your comments in green.

Minor comments:

Line 116: What is the classification of "nucleopolyhedrovirus"?

Response: In the revised version of the manuscript, we restructured this section of the Introduction to improve clarity and flow. As part of these revisions, the specific reference to nucleopolyhedrovirus was removed and the paragraph now provides a more general overview of previous studies on transgenerational immune priming in Spodoptera. Therefore, this term no longer appears in the manuscript.

Line 122: Could you provide the full name of SpexNPV and its classification?

Response: This section of the Introduction was restructured in the revised manuscript, and the reference to SpexNPV was removed.

General comments on the introduction: The concept of immune priming was well established and comprehensive. The authors introduce the methods, etc. However, we need some introduction to the pathogen used in the study. Why did the authors choose Metarhizium brunneum? What type of immune response is induced by the infection? Is the S. frugiperda-M. brunneum system a good model to study the immune system (natural infection?)?

Response: We rewrote the paragraph, and we included the information in the introduction related to the infection with the fungus. Lines 103-114, 132-154 and 159-174.

Line 202: No Figure S2 is attached to the manuscript.

Response: The reference to Figure S2 was included by mistake. We have removed this reference from the revised manuscript.

Lines 213-214: For the random pairing, were control and memory groups mixed, or did the authors mix males and females? This sentence could be clearer for the reader.

Response: We have clarified this sentence in the Methods to indicate that males and females were randomly paired within the same treatment group (memory × memory or control × control). Lines 202-206.

Line 298: Phenoloxidase—is this an assay, a measurement, or something else? Please clarify.

Response: We clarified this in the revised manuscript by specifying that phenoloxidase was measured using a phenoloxidase activity assay. Line 289-300.

Line 229: Same comment—could you clarify?

Response: As above, we clarified that this refers to a lytic activity assay, and the section title and description of the method were updated accordingly. Line 302-309.

© 2026. Published by The Company of Biologists under the terms of the Creative Commons Attribution License (https://creativecommons.org/licenses/by/4.0/).

Figure 2: The figure legend is scarce and incomplete for the reader to understand the figure. The authors need to provide a scale and a legend for the pictures (in both legend and figure). The authors need to provide a positive control for GFP—ideally a picture with GFP-positive infection, and then after clearance. That would be helpful.

Response: We revised the figure legend to provide a clearer and more detailed description of the images and included the magnification used. Lines 212-231.

Figure 3: Statistical analyses, while clearly stated in the results text, are missing in both the figure and legend. Please add these.

Response: Statistical information has been added to both the figure and the legend. The legend now specifies the statistical test used (log-rank test) to evaluate differences among survival curves. Lines 411-414, 439-512.

Lines 481-482: Reference the figure supporting this statement.

Response: We have referenced the figure. Line 477-486.

Figure 4: Like Figure 3, statistics are needed for Figure 4 in both the figure and the legend.
Response: Statistical information has been added to both the figure and the legend. Lines 439-512.

Lines 503-509: Authors need to reference the figure associated with the statistics.

Figure 5: The authors added the Kruskal-Wallis test statistics in the figure, but no details are given in the legend, so it is unclear what the test relates to. The legend needs to be more complete and descriptive, with colour references and statistics included.

Response: We have revised the legend of Figure 5 to provide a complete description. The legend now specifies the statistical tests performed (Kruskal–Wallis followed by pairwise Wilcoxon rank-sum tests with Bonferroni correction), clearly indicates that different letters represent significant differences among treatments within each panel, and includes sample sizes for all groups. Lines 514-553.

Line 552: Could the authors mention the values for phenoloxidase activity?

Response: The phenoloxidase analysis was performed using a GAMLSS model and pairwise comparisons of dispersion and mean, as reported in Tables S1–S2. The model focuses on the statistical differences among developmental stages and treatments rather than the absolute activity values, which were not calculated. Therefore, we report significance based on model-derived pairwise comparisons (p-values) rather than absolute mean ± SD. We have clarified this point in the text. Lines 564-578.

Figure 6: Statistics are needed in the figure and legend. Make the whiskers more visible. Describe the colours in the legend. Why did the authors not keep the colours used originally for each immunisation-challenge plan?
Response: Now, we report the statistics in the text and figure legends. We also homogenized the colors. Line 580-601.

Figure 7: Similar comments to Figure 6—see above.

Response: For Figures 6 and 7, we have included the statistical analyses in the figure legends, specifying the models used (Gamma GAMLSS) and pairwise comparisons with Tukey-adjusted p-

© 2026. Published by The Company of Biologists under the terms of the Creative Commons Attribution License (https://creativecommons.org/licenses/by/4.0/).

values. Whiskers have been thickened for better visibility, and the meaning of colors (pink for L5, purple for L6) is now explicitly described in the legend. Colors were chosen to represent the developmental stage at challenge and improve figure clarity, while treatments are shown along the x-axis. See lines 564-699.

Discussion: I really appreciated the discussion. The placement in the literature is good, but I would appreciate it if the authors could refer more to their own conclusions and observations. To help with that, I suggest that the authors reference figures in the discussion. For example, at line 662, the authors should reference their figure. The same applies to line 686 and each paragraph thereafter.

Response: We thank the reviewer for this suggestion. We have revised the Discussion to more explicitly link our interpretations with our results (for example see lines 726-756), and we have added references to the relevant figures throughout the section (i.e. line 739, 761, 781, 808 and 831).

<u>Second decision letter</u>

MS ID#: bio.062421R1

MS Title: Is the Developmental Stage Important for the Plasticity of Transgenerational Immune Priming?

Authors: Fernanda Vázquez-Fuerte; Angélica González-Hernández; Juan Carlos Torres Guzmán; Jorge Contreras-Garduño

I have now reached a decision on the above manuscript.

The reviewer reports are shown at the bottom of this email.

As you will see, the reviewers gave favourable reports, but raised a few critical points that will require amendments to your manuscript. In particular, the reviewers would like to see stronger evidence of the transgenerational immune-priming. If it is not possible to show additional data of immune markers, this should be addressed in the discussion as a potential limitation of the study. In addition, we would request that the title be changed so that it is not anthropomorphizing and better represents the findings here. Also, the last sentence in the caption for Fig. 1 needs to be edited. “Non offspring die as in panel C but only as in panel D.”

I hope that you will be able to carry out these changes, because we would like to be able to accept your paper. At this stage, we also ask you to ensure your manuscript complies with our formatting guidelines – please see our manuscript preparation guidelines for details. Provided you are able to fully address the referees’ comments, we are positive about publication of your paper (we accept over 95% of revision submissions) and therefore hope you won’t mind any extra work involved in reformatting your manuscript at this point.

Please upload both a 'clean' version of your Word file, along with a highlighted version clearly showing where you have made changes in the revised manuscript. Please avoid using 'Track changes' in Word files as these are lost in PDF conversion.

I should be grateful if you would also provide a point-by-point response detailing how you have dealt with the points raised by the reviewers in the 'Response to Reviewers' box. Please attend to all of the reviewers’ comments. If you do not agree with any of their criticisms or suggestions, please explain clearly why this is so.

© 2026. Published by The Company of Biologists under the terms of the Creative Commons Attribution License (https://creativecommons.org/licenses/by/4.0/).

Reviewer 1

Comments for the author

I appreciate the hard work that went into this revision, and y'all did a fantastic job! I did find a few more (very minor) things, all of which should be easy to fix!

Summary: In this paper by Vázquez-Fuerte et al., the possibility of immune memory passed down from one generation to another is discussed using Spodoptera frugiperda and infecting with the fungus, Metarhizium brunneum, in both the F0 generation and their offspring. They found that, if the parental generation was infected at the L5 stage, their offspring would have better survival if also challenged at the L5 stage compared to those whose parents were not infected, showing immune memory passed down from the parental generation. They also saw significant differences between immune markers at various stages, but not between treatments. Overall, this is fascinating work in physiology of insects that could have impacts in control methods in the future.

Experimental Quality: The experiments in this paper are robust and sound, and that has been made clearer with the revisions.

Reproducibility: Experiments performed in this paper seem reproducible, and sample sizes are appropriate.

Completeness: This paper is complete as is. I would personally like to see comparisons of the immune markers in the mixed-stage treatment groups (L5→L6; L6→L5; etc.), since I think it would add more information to this paper and would complement the current data. However, if the authors have other plans for that data, I do not want to hold this paper back for that data. The paper is complete without it if the authors do not wish to include that data here.

Scholarship: The authors discuss relevant literature in their field in order to put their work into context, both supporting and opposing.

Line-by-line:

Title: I LOVE a fun title, but I'm not sure I get the not wanting to grow up? Maybe I'm missing something obvious-whether you keep that or switch it, I think having a fun title is great!

Intro

Lines 112-114, 133-135: Please consider revising one of these, as they are basically the same sentence.

Methods

Line 299: Phenoloxidase should be one word.
Line 420: There is a lone "o" here-should it be "of?"

Results

Line 434: Subject-verb disagreement: should be "suggests."
Line 451: missing parentheses after Fig. 3

Discussion:

Great job with integrating your findings into the literature review!

Figures:

A lot of the figures are blurry on my end, like they are screenshots instead of PDFs. All the supplemental figures look great, but the main ones are blurry.

© 2026. Published by The Company of Biologists under the terms of the Creative Commons Attribution License (https://creativecommons.org/licenses/by/4.0/).

Figure 1: I really like the addition of this! However, the boxes look like they don't line up perfectly. (I know that's minor, but it should be an easy fix!)

Reviewer 2

Comments for the author

The authors carefully addressed all my comments; the manuscript is clearer, easier to follow, and the data are better presented.

One question remains: Did the authors observe immune protection in larvae from the immune-primed group or not? This is not shown, as the markers used here are not significantly different, as already noted in Reviewer 1's comment.

I would have suggested looking at the level of fungal infection to validate the function of immune priming ("transgenerational immune protection"). While this would be a valuable addition to the manuscript, I think the information currently presented is sufficient for publication.

Minor comments:

Line 95: the sentence may be slightly complex

Line 138: reference is incompletereference is incomplete

Repetition at lines 135-138 and lines 147-150

Line 152: the authors note that the offspring is challenged with an LD100; is that the same for all fungal challenges?

Reviewer's Responses to Questions

Experimental quality

Does each figure have the proper controls?
If 'No', please indicate reasons in Comments for Author box below.
Reviewer #1:
- Yes

Reviewer #2:
- Yes

Were the data analyzed using appropriate statistical tests?
If 'No', please indicate reasons in Comments for Author box below.
Reviewer #1:
- Yes

Reviewer #2:
- Yes

Reproducibility

Were experiments performed using adequate number of biological replicates?
If 'No', please indicate reasons in Comments for Author box below.
Reviewer #1:
- Yes

© 2026. Published by The Company of Biologists under the terms of the Creative Commons Attribution License (https://creativecommons.org/licenses/by/4.0/).

Reviewer #2:

- Yes

Does the methods section provide sufficient detail to permit reproducibility?
If 'No', please indicate reasons in Comments for Author box below.
Reviewer #1:

- Yes

Reviewer #2:

- Yes

Completeness

Are the manuscript's conclusions supported by the data?
If 'No', please indicate reasons in Comments for Author box below.
Reviewer #1:

- Yes

Reviewer #2:

- Yes

Scholarship
Do the authors cite and discuss the merits of data that would argue for and against their conclusion?
If 'No', please indicate reasons in Comments for Author box below.
Reviewer #1:

- Yes

Reviewer #2:

- Yes

Does the manuscript title & abstract accurately reflect the contents of the manuscript, without hyperbole?
If 'No', please indicate reasons in Comments for Author box below.
Reviewer #1:

- Yes

Reviewer #2:

- Yes

Second revision

Author response to reviewers' comments

We want to thank you and the reviewers for your constructive feedback on our manuscript number: **MS ID#: bio.062421R1** entitled: **"I don't want to grow up: the developmental stage may impair the transgenerational immune priming".** We have carefully considered each comment and made the necessary revisions accordingly. We decided to keep original title: **IS THE DEVELOPMENTAL STAGE IMPORTANT FOR THE PLASTICITY OF TRANSGENERATIONAL IMMUNE PRIMING?**

Below, we highlight in black the comments and our response in blue. In our revised manuscript, we also marked the changes incorporated based on suggestions in green.

© 2026. Published by The Company of Biologists under the terms of the Creative Commons Attribution License (https://creativecommons.org/licenses/by/4.0/).

We are open to carrying out more changes if needed.

Reviewer 1: I appreciate the hard work that went into this revision, and y'all did a fantastic job! I did find a few more (very minor) things, all of which should be easy to fix!

Summary: In this paper by Vázquez-Fuerte et al., the possibility of immune memory passed down from one generation to another is discussed using Spodoptera frugiperda and infecting with the fungus, Metarhizium brunneum, in both the F0 generation and their offspring. They found that, if the parental generation was infected at the L5 stage, their offspring would have better survival if also challenged at the L5 stage compared to those whose parents were not infected, showing immune memory passed down from the parental generation. They also saw significant differences between immune markers at various stages, but not between treatments. Overall, this is fascinating work in physiology of insects that could have impacts in control methods in the future.

Response: Thank you very much for your comments!

Completeness: This paper is complete as is. I would personally like to see comparisons of the immune markers in the mixed-stage treatment groups (L5→L6; L6→L5; etc.), since I think it would add more information to this paper and would complement the current data. However, if the authors have other plans for that data, I do not want to hold this paper back for that data. The paper is complete without it if the authors do not wish to include that data here.

Response: We thank the reviewer for this valuable suggestion. We agree that comparisons including the mixed developmental-stage groups (L5→L6 and L6→L5) provide additional insight into the influence of developmental stage on immune responses. These mixed-stage treatments were originally established primarily to evaluate survival following priming and challenge. Because survival after the second challenge was substantially reduced in the mixed-stage groups, only a limited number of individuals remained available for hemolymph collection and subsequent immune assays, resulting in smaller sample sizes than in the main experiment. Nevertheless, following the reviewer's suggestion, we analyzed all available samples and carry out additional experiments. The results are shown in Supplementary Material. The additional analyses support the conclusions of the main manuscript: although treatment strongly influenced survival outcomes, the mixed-stage treatments did not reveal clear differences in the immune parameters measured. Thus, these supplementary analyses reinforce, rather than modify, our overall conclusion that developmental stage is a key determinant of immune memory outcomes, whereas changes in immune marker activity do not fully explain the survival patterns observed. See also lines 265-279, 287-288, 290-304, 344-359, 377-393, 417-432, 455-468, and 615-635.

Title: I LOVE a fun title, but I'm not sure I get the not wanting to grow up? Maybe I'm missing something obvious-whether you keep that or switch it, I think having a fun title is great!

Response: Thank you for your comment. We have decided to retain the title: Is the Developmental Stage Important for the Plasticity of Transgenerational Immune Priming?

Intro

Lines 112-114, 133-135: Please consider revising one of these, as they are basically the same sentence.

Response: We deleted lines 112-114. This sentence is now in lines 111-116.

Line 299: Phenoloxidase should be one word.

Response: See the correction in lines 818, 847,

Line 420: There is a lone "o" here-should it be "of?"

Response: Corrected in line 968.

© 2026. Published by The Company of Biologists under the terms of the Creative Commons Attribution License (https://creativecommons.org/licenses/by/4.0/).

Results

Line 434: Subject-verb disagreement: should be "suggests."

Response: Done in line 125.

Line 451: missing parentheses after Fig. 3

Response: We included the parentheses in line 142.

Discussion:
Great job with integrating your findings into the literature review!

Response: Thank you very much.

Figures:
A lot of the figures are blurry on my end, like they are screenshots instead of PDFs. All the supplemental figures look great, but the main ones are blurry.

Response: Thank you for pointing this out. We have replaced all the main figures with higher-resolution versions to improve their clarity and readability. Also, the updated figures have been uploaded in the appropriate format and should now display correctly in the revised manuscript.

Figure 1: I really like the addition of this! However, the boxes look like they don't line up perfectly. (I know that's minor, but it should be an easy fix!)

Response: Thank you so much. We improved this figure.

Reviewer 2: The authors carefully addressed all my comments; the manuscript is clearer, easier to follow, and the data are better presented.

One question remains: Did the authors observe immune protection in larvae from the immune-primed group or not? This is not shown, as the markers used here are not significantly different, as already noted in Reviewer 1's comment.
I would have suggested looking at the level of fungal infection to validate the function of immune priming ("transgenerational immune protection"). While this would be a valuable addition to the manuscript, I think the information currently presented is sufficient for publication.

Response: We thank the reviewer for this important comment. In this study, evidence of immune protection was evaluated based on the survival outcome following immune priming and subsequent challenge, rather than solely on the immune markers measured. Although we did not observe significant differences in the humoral and oxidative immune parameters evaluated in the offspring, the survival response provides functional evidence of altered host resistance after immune priming. We agree that quantifying fungal burden or pathogen growth after challenge would provide an additional and valuable measure to confirm the functional mechanisms underlying transgenerational immune protection. We have now acknowledged this limitation in the Discussion and clarified that the immune parameters analyzed here represent potential physiological correlates of immune priming, while future studies should incorporate pathogen load measurements to directly assess infection control.

Minor comments:

Line 95: the sentence may be slightly complex

Response: We improved this sentence. Lines 65-68.

Line 138: reference is incomplete

© 2026. Published by The Company of Biologists under the terms of the Creative Commons Attribution License (https://creativecommons.org/licenses/by/4.0/).

Response: Thanks, we inserted the reference in line 111.

Repetition at lines 135-138 and lines 147-150

Response: Thanks. This information was corrected in line 111-116.

Line 152: the authors note that the offspring is challenged with an LD100; is that the same for all fungal challenges?

Response: Thank you for this observation. In the experiment described in this section, offspring were challenged exclusively with the Cherry strain of *Metarhizium brunneum*. Therefore, the LD100 dose mentioned here (2,000 conidia/μL) corresponds specifically to the Cherry strain. We have clarified this point in the Methods section.

Third decision letter

MS ID#: bio.062421R2

MS Title: Is the Developmental Stage Important for the Plasticity of Transgenerational Immune Priming?

Authors: Fernanda Vázquez-Fuerte; Angélica González-Hernández; Juan Carlos Torres Guzmán; Jorge Contreras-Garduño

I am happy to tell you that your manuscript has been accepted for publication in Biology Open, pending our standard publication integrity checks. It was accepted on 16th July 2026.

© 2026. Published by The Company of Biologists under the terms of the Creative Commons Attribution License (https://creativecommons.org/licenses/by/4.0/).
